# Real-World Implementation and Evaluation of AI-Driven Clinical Decision Support in Emergency Medicine: A Systematic Review

**DOI:** 10.3390/healthcare14182922

**Published:** 2026-09-09

**Authors:** Mohammad Saleem, Mahdieh Zare Bidoki, Wafa Alsuraihi, Mohammed Ali Al-Garadi, Abdulaziz Ahmed

**Affiliations:** 1 Department of Health Services Administration, School of Health Professions, University of Alabama at Birmingham, Birmingham, AL 35233, USA; saleem@uab.edu (M.S.); mzare@uab.edu (M.Z.B.); 2 Department of Business Administration, University of Fairfax, Salem, VA 24153, USA; alsuraihiw@students.ufairfax.edu; 3 Department of Biomedical Informatics, Vanderbilt University Medical Center, Nashville, TN 37203, USA; mohammed.a.al-garadi@vumc.org; 4 Department of Biomedical Informatics and Data Science, Heersink School of Medicine, University of Alabama at Birmingham, Birmingham, AL 35233, USA

**Keywords:** artificial intelligence, clinical decision support systems, emergency department, real-world implementation, predictive modeling, systematic review

## Abstract

**Background/Objectives:** Emergency departments (EDs) are high-pressure environments where time-sensitive decisions, fragmented data, and operational strain create strong demand for AI-driven clinical decision-support systems (AI-CDSSs). These systems have shown promise in triage, diagnosis, risk stratification, and workflow optimization, yet real-world implementation in emergency medicine remains uneven. This systematic review aimed to synthesize the technical characteristics, clinical applications, implementation dimensions, organizational and ethical considerations, and real-world impact of AI-CDSSs in ED settings. **Methods:** This systematic review followed PRISMA guidance and searched PubMed, Scopus, and Embase for English-language studies published between January 2015 and February 2025. Eligible studies described AI-CDSS implementation, clinical integration, or performance evaluation in ED settings. Twenty-three studies met the inclusion criteria and were synthesized across five domains: technical characteristics, clinical applications, implementation dimensions, organizational and ethical considerations, and real-world impact. **Results:** Among the 23 included studies, 20 contributed to the real-world evaluation synthesis. Of these 20 studies, 8 (40%) achieved live or prospective evaluation, seven (35%) relied only on retrospective validation, four (20%) used human-centered or perception-based evaluation, two (10%) used post-implementation assessment, and one (5%) used simulation-based evaluation; categories were not mutually exclusive because some studies employed more than one evaluation approach. In the separate clinical-application synthesis of 20 studies, AI-CDSS were most frequently applied to diagnosis and immediate intervention (45%), followed by prediction and risk stratification (35%) and operational improvement (20%). Successful adoption was more consistently associated with EHR integration, workflow-sensitive design, and clinician engagement than with algorithmic performance alone. Persistent barriers included limited external validation, weak drift-monitoring plans, inconsistent usability testing, regulatory ambiguity, and insufficient equity mitigation. **Conclusions:** Sustainable implementation of AI-CDSSs in emergency medicine will require prospective multi-site evaluation, sociotechnical integration, adaptive governance, and greater attention to equity. Technical performance alone is insufficient to establish clinical readiness; successful implementation also depends on integration with clinical workflows, clinician engagement, ongoing monitoring, and appropriate governance.

## 1. Introduction

The emergency department (ED) represents one of the most challenging healthcare environments, characterized by time constraints, high-stakes decision-making, and a diverse patient population presenting with various acuity levels [1,2]. In the United States, EDs manage approximately 155 million patient visits annually, translating to an average of 47 visits per 100 people [3], with a 31.5% increase in visit rates observed over the past two decades [4]. In 2017, there were over 25 million injury-related ED visits in the United States, highlighting the ED’s role in acute trauma care [5]. Approximately 13.1% of ED visits resulted in hospital admissions, underscoring the severity of conditions managed [4]. In England, a recent medRxiv preprint reported approximately 14 million annual visits to major EDs, with admission rates of approximately 20% [6]. Similarly, in Australia, ED presentations have continued to grow. In Victoria alone, public hospital EDs managed approximately 1.9 million presentations annually [7]. This surge in utilization, coupled with the increasing complexity of cases, has placed unprecedented strain on ED care systems worldwide.

AI-CDSSs in emergency medicine draw on several distinct methodological families, each with different implications for deployment. Conventional supervised machine learning uses regression, tree ensembles such as Random Forests, gradient boosting and their variants and dominates ED risk prediction because it performs well on structured triage vitals, laboratory values and administrative data, trains on cohorts of the size a single hospital can assemble, and yields feature-importance measures that clinicians can inspect. Deep neural networks extend this to waveform and image data, supporting electrocardiogram interpretation and radiological triage, at the cost of larger training requirements and reduced transparency. Transformer-based and pre-trained language models, including domain-adapted encoders such as BioClinicalBERT, allow unstructured triage notes and narrative documentation to be used as model input, and generative large language models are now being investigated for documentation assistance, differential-diagnosis generation and patient communication, although prospective ED evaluations remain scarce. Predictive analytics applied at the level of the department rather than the patient queueing, patient-flow and demand-forecasting models target crowding and resource allocation. Cutting across all of these, explainable AI methods are either inherent to the model class (interpretable coefficients, decision trees, Bayesian networks, ontology-based reasoning) or applied post hoc to an opaque model (SHAP, LIME, saliency and attention visualization).

These families map onto four broad functions in the ED. In triage, models stratify acuity and predict admission, intensive care requirement or deterioration, supporting the allocation of scarce beds and clinician attention. In diagnosis, they support detection of time-critical conditions such as acute coronary syndrome, sepsis and stroke, where earlier recognition changes management. In workflow optimization, they forecast arrivals, predict length of stay and prioritize imaging and laboratory ordering. In patient safety, they act as surveillance layers that detect deterioration, flag medication and documentation errors, and reduce missed diagnoses. The methodological family adopted therefore constrains not only accuracy but also interpretability, data requirements, maintenance burden and ultimately the plausibility of sustained clinical use, which is why this review examines technical characteristics and implementation outcomes together rather than separately.

In response to these pressures, artificial intelligence (AI) has emerged as a transformative force in healthcare, enabling the development of advanced clinical decision-support systems (CDSSs) that enhance clinical efficiency and decision accuracy [8]. Recent surveys indicate that while 63% of healthcare organizations are implementing AI solutions and EDs have been at the forefront of this adoption [9]. In emergency medicine, AI-based CDSSs are increasingly used for key functions such as triage, diagnostic support, risk stratification, treatment recommendation, resource allocation, and documentation assistance. For example, machine learning (ML)-based triage systems have demonstrated 15–20% reductions in waiting times for critically ill patients through real-time predictive analytics [10]. Similarly, ML-driven diagnostic and risk stratification models have reduced error rates and achieved sensitivities and specificities above 85% for critical outcomes. These advances illustrate the capacity of AI not only to enhance decision-making under time constraints but also to improve throughput and safety in high-acuity environments such as the ED.

The reach of AI in healthcare now extends well beyond bedside decision support. Network-based computational methods are used to prioritize disease-associated genes and elucidate mechanisms in heterogeneous complex disease, for example through dual ranking over multiplex biological networks [11], while related approaches support drug repositioning, medical image interpretation, genomic variant classification and epidemiological forecasting. This breadth matters for emergency medicine in two ways. It indicates that the underlying methodological advances are general rather than specialty-specific, so that progress in adjacent biomedical domains transfers to acute care; and it highlights, by contrast, how much of the emergency-medicine literature remains at the model-development stage, since the translational and governance requirements of a time-critical clinical environment are more demanding than those of retrospective computational analysis.

The interpretability of AI-CDSSs differs in kind, not merely in degree, from that of the clinical scores they are proposed to replace. A traditional score such as CURB-65, the Wells criteria or HEART is transparent in a specific and demanding sense: it uses a small number of clinically meaningful variables, combines them by arithmetic the clinician can perform mentally, and was derived in a manner the clinician can inspect and contest. The physician can therefore reconstruct the entire inferential path from patient to recommendation, recognize when a particular patient falls outside the score’s assumptions, and justify a departure from it. Machine learning models sever this chain. A gradient boosting ensemble over several hundred features, or a transformer over free-text triage notes, computes a decision surface that cannot be reconstructed by a clinician at the bedside irrespective of that clinician’s expertise, because the limiting factor is representational complexity rather than training.

Explainable AI methods are frequently presented as closing this gap, but they close it only partially, and the distinction matters for how clinicians perceive these systems. Post hoc techniques such as SHAP and LIME produce a locally faithful approximation of the model’s behavior around a single prediction; they report which features moved the output and in which direction, but not the mechanism by which the model related them, and different explanation methods applied to the same model can yield different attributions. What the clinician receives is therefore a plausible account of the output rather than the reasoning that produced it. This has two consequences documented in the studies reviewed here. First, plausible explanations can increase confidence without increasing correctness, since an explanation that accords with clinical intuition is persuasive whether or not the underlying prediction is sound—a concern that has motivated arguments for using inherently interpretable models in high-stakes settings rather than explaining opaque ones after the fact. Second, the burden of interpretation falls unevenly: as Piñero de Plaza et al. [12] observed across 12 EDs, less experienced clinicians reported greater difficulty interpreting model outputs, so explanation facilities that assume a sophisticated user may widen rather than narrow disparities in effective use. For the emergency physician, the practical question is not whether a model is explainable in principle but whether, in the seconds available during a resuscitation, its output can be appraised, trusted or overridden with justification. This is why we treat explainability throughout this review as an implementation property inseparable from training, workflow position and accountability, rather than as a technical attribute of the model alone.

Despite these promising applications, a comprehensive understanding of the real-world implementation, effectiveness, limitations, and clinical integration of AI-CDSSs in ED settings remains limited. Prior reviews have largely focused on specific facets of AI rather than its translation into practice. For example, Boonstra et al. [13] examined how AI technologies influence clinician work design but did not assess clinical performance or implementation feasibility. Szymczyk et al. [14] summarized 59 studies published between 2020 and 2023, emphasizing diagnostic accuracy and sensitivity while omitting sociotechnical and governance challenges. Kareemi et al. [15] conducted a large scoping review that mapped the developmental stages of AI-CDSS tools but intentionally excluded post-implementation and workflow integration analyses. These reviews show that the literature remains stronger in evaluating technical capability than in explaining whether AI-CDSS can be integrated into emergency care in a sustainable, trustworthy, and clinically meaningful manner.

Building upon these foundations, this systematic review addresses three review questions (RQ): RQ1—what is the current state of technical performance and validation of AI-CDSSs deployed or evaluated in ED settings, including algorithmic foundations, discrimination metrics, external validation, and explainability? RQ2—what implementation outcomes including EHR integration, clinician adoption, usability, stakeholder engagement, and workflow fit, have been reported for AI-CDSS in real-world ED contexts, and what barriers and enablers shape these outcomes? RQ3—what organizational, ethical, regulatory, and equity considerations have been identified in relation to AI-CDSS deployment in emergency medicine, and how do these influence sustainable, equitable implementation? The population of interest is clinicians and patients in ED settings; the concept encompasses AI-CDSS design, integration, and evaluation; and the context spans academic, community, and resource-variable EDs across international settings. By addressing these questions, the review aims to provide actionable insights for clinicians, informaticians, administrators, and policymakers seeking to translate AI innovations into sustainable emergency care practice.

## 2. Materials and Methods

### 2.1. Search Strategy

We conducted this systematic review in accordance with the PRISMA guidelines. The completed PRISMA 2020 checklist is provided in File S1. A comprehensive search of PubMed, Scopus, and Embase identified studies published between January 2015 and February 2025. The search strategy combined free-text terms with controlled vocabulary. PubMed searches included the MeSH headings “Emergency Service, Hospital,” “Artificial Intelligence,” and “Decision Support Systems, Clinical, while Embase searches used the corresponding Emtree terms “hospital emergency service,” “artificial intelligence,” and “clinical decision support system”. Implementation-related terminology was additionally captured using free-text terms. Database-specific syntax was adapted for each database, and the complete search strategies are provided in File S2.

Four core concepts guided the query design: (1) implementation, (2) artificial intelligence and machine learning techniques (including deep learning, natural language processing (NLP), and predictive modeling), (3) decision-support systems, and (4) emergency department or acute care settings. Boolean operators and truncation (e.g., “decision-support-system*”) captured variations in terminology. We conducted the searches in February 2025. The search results were imported into Covidence, which automatically identified duplicates before manual screening. Two reviewers independently applied the eligibility criteria, resolving disagreements by consensus. The search retrieved 52 records from PubMed, 46 from Scopus, and 96 from Embase prior to deduplication. File S2 provides the complete database-specific queries, including Boolean operators and filters, to support reproducibility.

### 2.2. Inclusion and Exclusion Criteria

#### 2.2.1. Inclusion Criteria

Studies were eligible for inclusion if they met the following criteria: they were (i) published between January 2015 and February 2025; (ii) written in English; (iii) original, peer-reviewed primary research articles; (iv) focused on the implementation of CDSS in ED settings; (v) employed AI, ML, deep learning (DL), or predictive modeling techniques; and (vi) reported on implementation processes, clinical integration, or performance evaluation.

#### 2.2.2. Exclusion Criteria

Studies were excluded if they were (i) review papers, (ii) editorials, (iii) opinion pieces, (iv) letters, or (v) conference abstracts without full-text availability.

### 2.3. Study Selection

**Figure 1:**
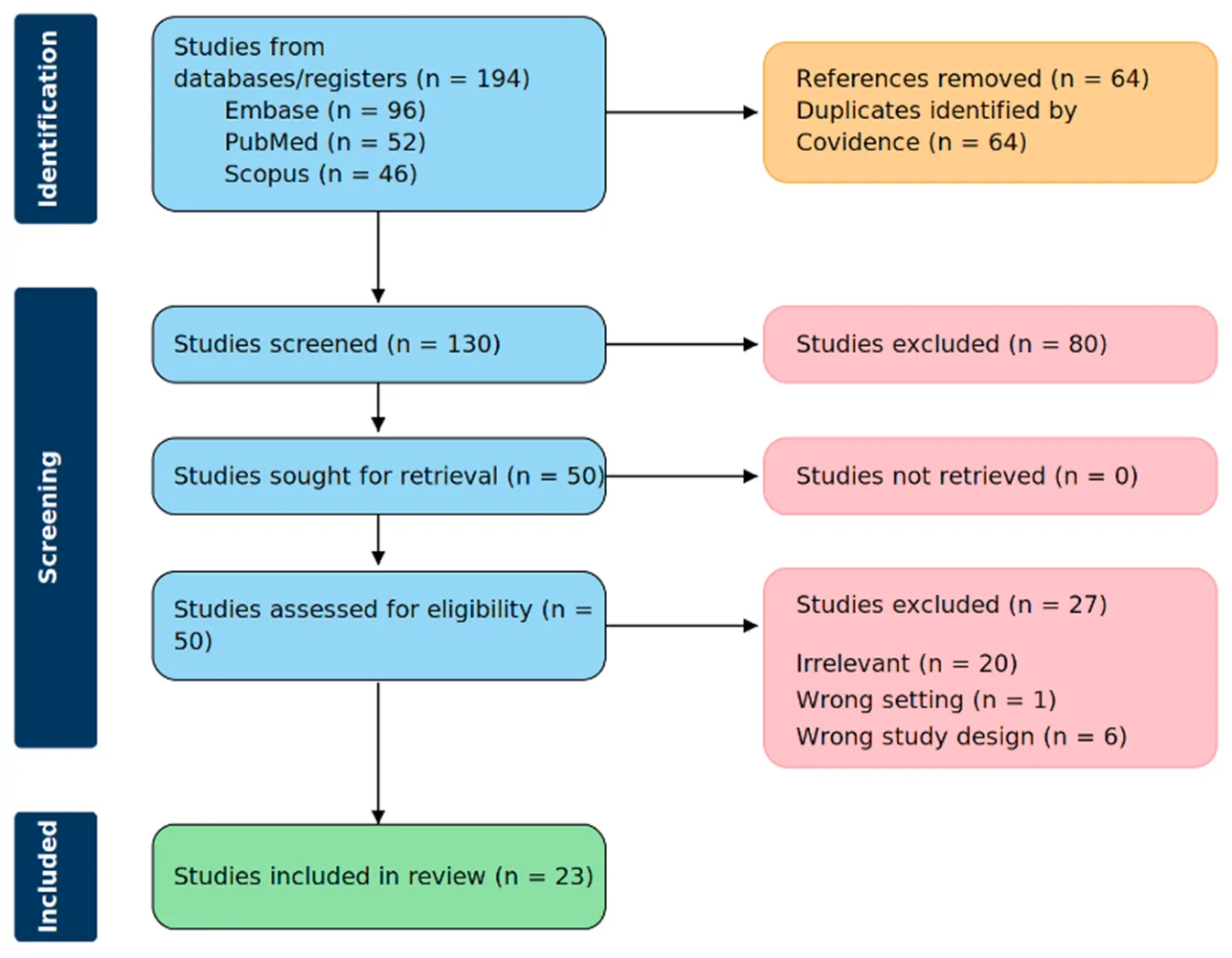
PRISMA flow diagram of the study selection process.

A total of 194 studies were initially identified and imported into Covidence (Veritas Health Innovation, Melbourne, Australia; available at www.covidence.org) for screening. After removing 64 duplicates, 130 unique records remained for title and abstract screening; 80 were excluded as irrelevant. Two reviewers assessed the remaining 50 full-text articles in detail, resulting in the exclusion of 27 studies due to irrelevance (20), inappropriate study design (6), or incorrect setting (1). Ultimately, 23 studies met all inclusion criteria and were included in the final systematic review. Figure 1 presents the PRISMA flow diagram for the study selection process.

### 2.4. Data Extraction and Analysis

Data extraction and synthesis followed a structured, multi-step approach designed to capture both the technical characteristics and contextual implementation features of AI-CDSSs within ED settings.

In the first step, an initial review of all eligible full-text articles identified recurring concepts, methodological patterns, and system characteristics. This inductive assessment informed the development of a thematic framework comprising five overarching domains: (i) Technical Characteristics and Performance Metrics of AI-CDSSs; (ii) Clinical Applications and Use Cases; (iii) AI-CDSS Implementation Dimensions; (iv) Organizational, Policy, and Ethical Considerations; and (v) Real-World Impact and Evaluation.

In the second step, a standardized template aligned with the five domains guided detailed data extraction from each included study. Technical variables included algorithmic foundations (e.g., logistic regression, decision trees, Random Forests, convolutional neural networks), model performance indicators (e.g., area under the receiver operating characteristic curve (AUROC), sensitivity, specificity, accuracy), data types and sources (e.g., structured EHR data, unstructured clinical notes, imaging data, real-time physiological inputs), validation approaches (e.g., internal cross-validation, external testing, prospective evaluation), and explainability tools (e.g., Shapley Additive Explanations (SHAP), Local Interpretable Model-Agnostic Explanations (LIME), attention heatmaps). Additional technical variables included model inputs and modalities, model architecture/configuration, training or derivation dataset characteristics, calibration methods, uncertainty estimation, and computational or deployment requirements; Supplementary Table S1 provides the detailed study-level findings.

Digital-infrastructure variables included EHR/EMR/RIS/LIS integration, FHIR/HL7 interoperability, data harmonization or standardization, real-time data acquisition, cybersecurity, data-governance, and vendor/platform-related implementation issues; Supplementary Table S2 provides the study-level findings. Equity-related variables included demographic subgroup representation and performance, socioeconomic factors, racial and ethnic bias assessment, geographic variability, dataset representativeness, formal fairness metrics, and reported bias-mitigation strategies; Supplementary Table S3 provides the detailed study-level findings. Implementation-stage coding classified systems as development-only, pilot-tested, deployed in clinical workflow, or under ongoing maintenance based on explicit descriptions in each study.

**Figure 2:**
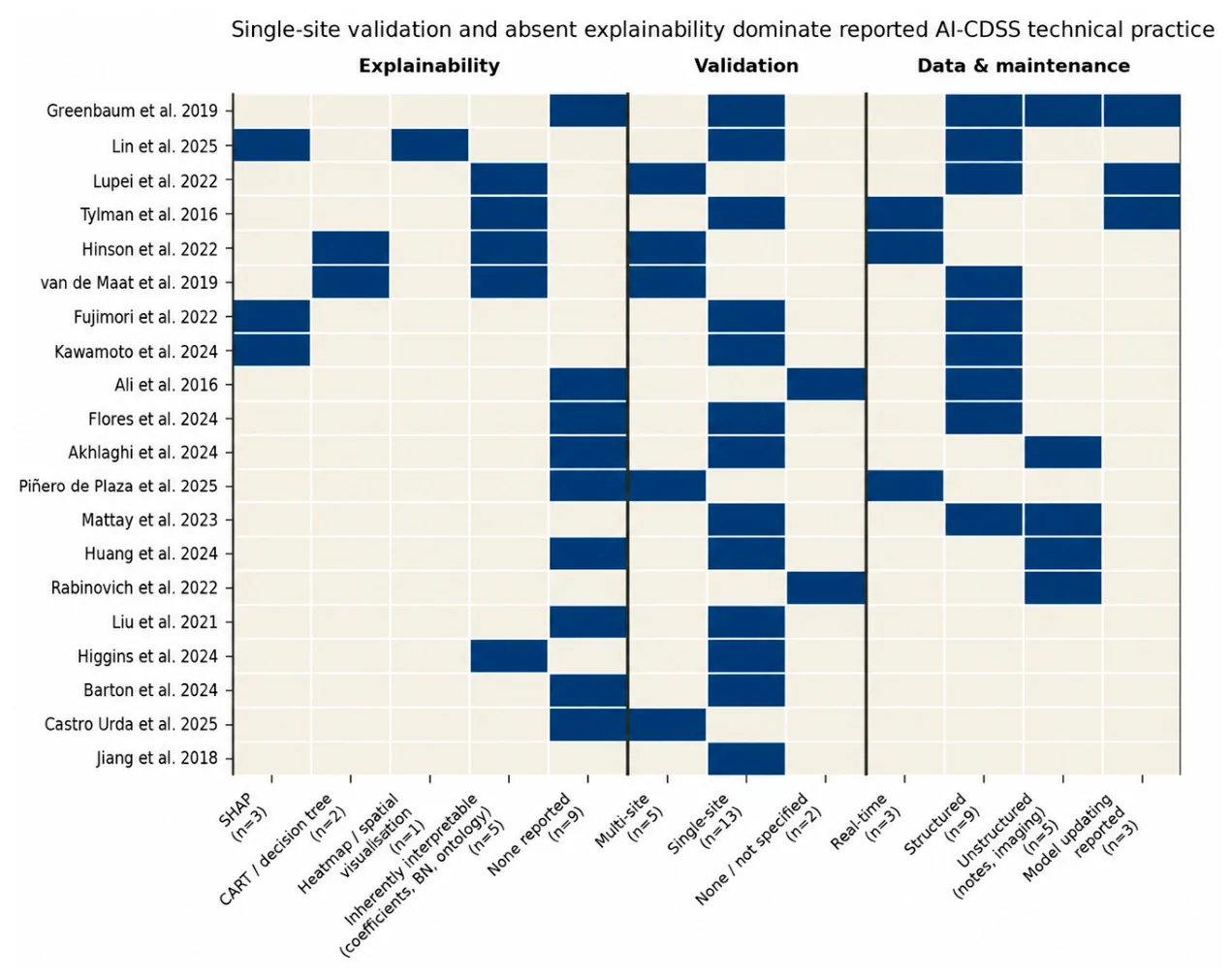
Reported technical features of AI-CDSS studies in emergency departments. Cells indicate whether each feature was reported for a given study (dark) or not (light); all cells are derived from the study characteristics in Table 1. Performance metrics are not shown, as reported metrics were heterogeneous and not comparable across studies; study-level metrics as reported are given in Supplementary Table S5 [12,16,17,18,19,20,21,22,23,24,25,26,27,28,29,30,31,32,33,34].

In the third step, narrative synthesis organized the extracted data according to the predefined thematic framework, enabling cross-study comparisons and identification of patterns in AI-CDSS maturity, technical sophistication, and deployment environments. Summary matrices further supported transparency and reproducibility. Because not all included studies provided sufficient information for every thematic domain, the analyses used domain-specific subsets. Studies contributed only to domains for which they reported relevant technical, clinical-application, implementation, organizational/policy/ethical, or evaluation information. Supplementary Table S4 maps all 23 included publications to each domain and provides the reasons for any domain-specific non-contribution. Accordingly, the technical synthesis and Figure 2 include 20 of 23 studies, the clinical-application synthesis includes 20 of 23 studies, the implementation synthesis includes 18 studies, and the real-world evaluation synthesis includes 20 of 23 studies. The relevant denominator is also identified in each corresponding results section.

**Table 1:** Technical Characteristics and performance metrics of AI-CDSS studies in emergency departments.

Study	Domain	Technical & Algorithmic Foundations	Model Performance (AUROC/Sens/Spec)	External Validation & Generalizability	Model Updating & Maintenance	Explainability & Transparency	Data Quality & Source
Higgins et al. [22]	Prediction and risk stratification	RF, XGB, CatBoost, kNN, EBM (InterpretML), SVM	EBM: 0.717/0.108/0.981; RF: 0.682/0.115/0.965	Single site; no external validation	Maintenance not specified	EBM interpretable; SHAP proposed	Retrospective (2016–2021), 2 EDs, Australia
Flores et al. [21]	Diagnosis and immediate intervention	RF, NN	RF: 0.80; NN: 0.77	Single-site validation	None	No SHAP/LIME	Structured LIS + EMR data
Lupei et al. [19]	Prediction and risk stratification	Logistic regression (LASSO-logit)	Temporal AUROC 0.87; Real-time 0.85	Multi-site (12 hospitals, same system)	Calibration issues; update implied	Interpretable coefficients	Structured EHR, 12 hospitals
Tylman et al. [28]	Prediction and risk stratification	Bayesian network, ECG segmentation	Sens 0.53–0.68; Spec 0.9–1.0	29 real patient cases	Auto-adjust via physician input	BN probabilities interpretable	Real-time vitals data
Barton et al. [33]	Prediction and risk stratification	ML-based fall-risk prediction algorithm	Not specified in implementation study	Single academic medical center (Wisconsin)	None	None discussed in implementation study	EHR data for patients ≥65 years
Greenbaum et al. [27]	Operational enhancement	HaPPy ontology, multiclass SVM	Best-5 accuracy 0.757	Single tertiary center	Iterative ontology refinement	Not detailed	Mixed structured + notes
Kawamoto et al. [26]	Diagnosis and immediate intervention	XGBoost	AUROC 0.72–0.78; Sens/Spec ~74%	Single cohort	None	SHAP for feature importance	Structured hospital data (2009–2015)
Jiang et al. [34]	Operational enhancement	Deep neural network	RMSE, MAPE (not stated)	Single-site	None	GA feature selection	Admin data, Hong Kong ED
Hinson et al. [24]	Operational enhancement	RF, CART	AUROC 0.91→0.85; 0.89→0.80	Single health system	None	Decision trees interpretable	Real-time EHR (vitals, labs)
Ali et al. [20]	Prediction and risk stratification	kNN, geospatial (SaTScan)	NRMSE 0.29	None	None	None	Mixed structured + geospatial
Lin et al. [18]	Diagnosis and immediate intervention	LDA, LR, RF, GBM, AdaBoost, XGB, LGBM	LGBM best; AUROC top in all 5 metrics	Limited; multicenter needed	None	SHAP + heatmaps	Structured CBC + DIFF, 2023
van de Maat et al. [29]	Diagnosis and immediate intervention	Logistic regression, CART	AUROC, ORC; Sens/Spec reported	Dual-site (NL + UK)	None	CART transparent; LR interpretable	Prospective structured data
Akhlaghi et al. [16]	Prediction and risk stratification	NLP-based ML	Sens 73.1%; Spec 74.3%; Acc 74%	Single-site	None	Binary outputs; no rationale	10-year triage notes
Piñero de Plaza et al. [12]	Diagnosis and immediate intervention	RAPIDx AI (black-box)	Human-centered eval only	Multi-site (12 EDs)	None	Numeric outputs only	Real-time clinical + labs
Mattay et al. [30]	Operational enhancement	Predictive text AI module	Unscored orders ↓81%→45%; +24 s alert time	Single quaternary ED	None	Ranked shortlist; no SHAP	Mixed text + structured data
Huang et al. [17]	Prediction and risk stratification	NLP, ML/DL, BioClinicBERT variants	AUROC 0.90–0.92 across tasks	Single hospital (Taiwan)	None	None	Mixed EHR + narratives
Fujimori et al. [23]	Diagnosis and immediate intervention	XGBoost (5-fold CV)	AUROC 0.901 (95% CI 0.84–0.96)	Small sample; simulated	None	SHAP bar charts	Structured EHR (27,550 pts, Japan)
Liu et al. [25]	Diagnosis and immediate intervention	Deep learning	F1: AMI 0.85; STEMI 0.93; NSTEMI 0.70	Single academic center	None	None	Mixed ECG + vitals + lab s
Rabinovich et al. [32]	Diagnosis and immediate intervention	AI for chest X-ray interpretation	Not specified	Not specified	None	Focus on clinician trust evaluation	Radiological imaging data
Castro Urda et al. [31]	Diagnosis and immediate intervention	AI-based Intelligen-C strategy for HCV screening	Not specified	Single health system	None	None	EHR data for automated HCV screening

↓ = decrease.

### 2.5. Quality Appraisal and Risk of Bias

We did not apply any structured quality or risk-of-bias instrument to the included studies. The review used descriptive synthesis to map technical, implementation, organizational, policy, ethical, and evaluation characteristics across heterogeneous study designs rather than formally grading study quality or pooling effect estimates.

## 3. Results and Discussion

### 3.1. Technical Characteristics and Performance Metrics of AI-CDSS Studies

The real-world implementation of AI-CDSSs in EDs reveals a fundamental mismatch between algorithmic ambition and implementation rigor that the field must resolve before these tools can be trusted in routine emergency care. As Table 1 shows, AI-CDSS models in EDs have become more technically sophisticated, but the methods used to validate, report, and maintain them have not advanced at the same pace. As a result, strong performance (e.g., AUROC) values often reflect good performance within a study rather than true readiness for safe and reliable clinical use. This section examines the extent to which the technical performance reported in AI-CDSS studies is accompanied by interpretability, external validation, maintenance planning, and clinical applicability in emergency department settings. Of the 23 included studies, 20 provided sufficient study-specific technical information to contribute to the technical synthesis and Figure 2 (Supplementary Table S4).

#### 3.1.1. Algorithmic Foundations and Performance

AI-CDSS studies in emergency departments used a broad range of algorithms, reflecting both the complexity of ED decision-making and the practical constraints of real-time care. As summarized in Table 1, Random Forest (RF) was the most frequently used algorithm, appearing in studies by [16,17,18,19]. XGBoost appeared in three studies [17,20,21], while logistic regression was used in at least three [18,19,22]. Advanced language models, including BERT, BLSTM, and GatorTron, were incorporated by Higgins et al. [22] and Fujimori et al. [23], indicating an emerging use of deep contextual NLP embeddings. These algorithmic choices suggest that most studies selected models that balance predictive accuracy with manageable computational demands, particularly for outcomes such as sepsis [18], infection [21,24], and mental health admission under real-time constraints [22]. This pattern is also reflected in the reported performance metrics. As shown in Table 1, Random Forest and logistic regression achieved AUROC values of 0.80 or higher in several studies, whereas XGBoost demonstrated more heterogeneous performance across applications, with AUROC values ranging from 0.72 to 0.90 depending on the clinical task and dataset. A smaller subset of studies indicated that deep learning approaches may provide additional advantages when the clinical task requires modeling more complex nonlinear, temporal, or multimodal relationships. For example, Liu et al. [25] applied a deep convolutional network to detect acute myocardial infarction (AMI), while Huang et al. [17] employed a transformer-based language model (BioClinicalBERT) integrating physiological and narrative data to predict multiple emergency interventions, both achieving AUROC values above 90%.

Among the studies that reported a threshold-independent measure of discrimination, AUROC was the metric of choice, but sensitivity–specificity trade-offs often revealed more clinically meaningful priorities. For performance evaluation, AUROC was the most frequently reported benchmark, but sensitivity–specificity trade-offs often revealed more clinically meaningful priorities. For instance, Higgins et al. [22] optimized for high specificity (98%) at the expense of sensitivity in mental health triage, highlighting a decision to reduce false positives. In contrast, infection and sepsis prediction models targeted more balanced operating thresholds to mitigate false negatives and enhance safety [18,26]. Other studies emphasized precision and positive predictive value when evaluating model performance. Akhlaghi et al. [16] reported these metrics in their NLP-based triage tool, while Liu et al. [25] highlighted the relevance of F1 scores in the context of pronounced class imbalance. These differences suggest that model performance in ED settings cannot be fully understood through AUROC alone, because the clinical consequences of error vary across use cases.

Reported performance metrics were markedly heterogeneous, which constrains synthesis. Of the 20 studies evaluating a model, only eight reported an area under the receiver operating characteristic curve. The remainder reported F1 scores for rhythm and infarct classification [25], normalized root mean squared error for outbreak forecasting [20], top-k accuracy for differential generation [27], sensitivity and specificity without a threshold-independent measure [28], ordinal c-statistics [29], or process and economic endpoints such as order-scoring completeness [30] and cost per Quality-Adjusted Life Year [31]. Two studies reported no model performance metric, having evaluated clinician perception or human-centered implementation outcomes instead [12,32]. Because target conditions, outcome prevalence and metric definitions differ across studies, these values are not commensurable and we do not pool or rank them; study-level metrics as reported are given in Supplementary Table S5.

This broader view of performance was also reflected in studies that evaluated workflow impact in addition to classification metrics. Mattay et al. [30] linked predictive text to a reduction in unscored imaging orders from 81% to 45% with modest alert-handling time, while Greenbaum et al. [27] achieved substantial documentation efficiencies through an ontology-based ML-driven interface. Barton et al. [33] used academic detailing to identify implementation barriers and educate clinicians after deployment, supporting workflow fit rather than reporting keystroke metrics. Pinero de Plaza et al. [12] further incorporated user-centered evaluation in the RAPIDx AI emergency cardiac care decision-support system trial, underscoring that clinical value depends not only on predictive performance but also on process efficiency, usability, and trust. Overall, the literature suggests that progress in AI-CDSS development for emergency departments has not been matched by comparable advances in external validation, lifecycle maintenance, or transparency.

Figure 2 complements Table 1 by providing a cross-study visual summary of key methodological and implementation-relevant features in AI-CDSS research conducted in emergency departments. Rather than focusing on predictive performance alone, the heatmap maps whether individual studies incorporated explainability methods, implementation frameworks, external or multi-site validation strategies, real-time data streams, structured or unstructured data sources, and any attention to model monitoring or drift. This visualization highlights marked heterogeneity across the literature. While several studies reported strong AUROC values, these performance results often coexisted with limited external validation, sparse maintenance planning, and inconsistent transparency practices. Figure 2 also suggests that studies drawing on richer and more clinically integrated data sources were often more technically sophisticated, yet they still frequently lacked the broader methodological safeguards needed for routine implementation. Accordingly, the heatmap supports the broader conclusion of this section that reported discrimination performance should be interpreted alongside explainability, validation design, and lifecycle management, rather than treated as a standalone indicator of clinical readiness.

#### 3.1.2. Validation, Maintenance, and Transparency

**Figure 3:**
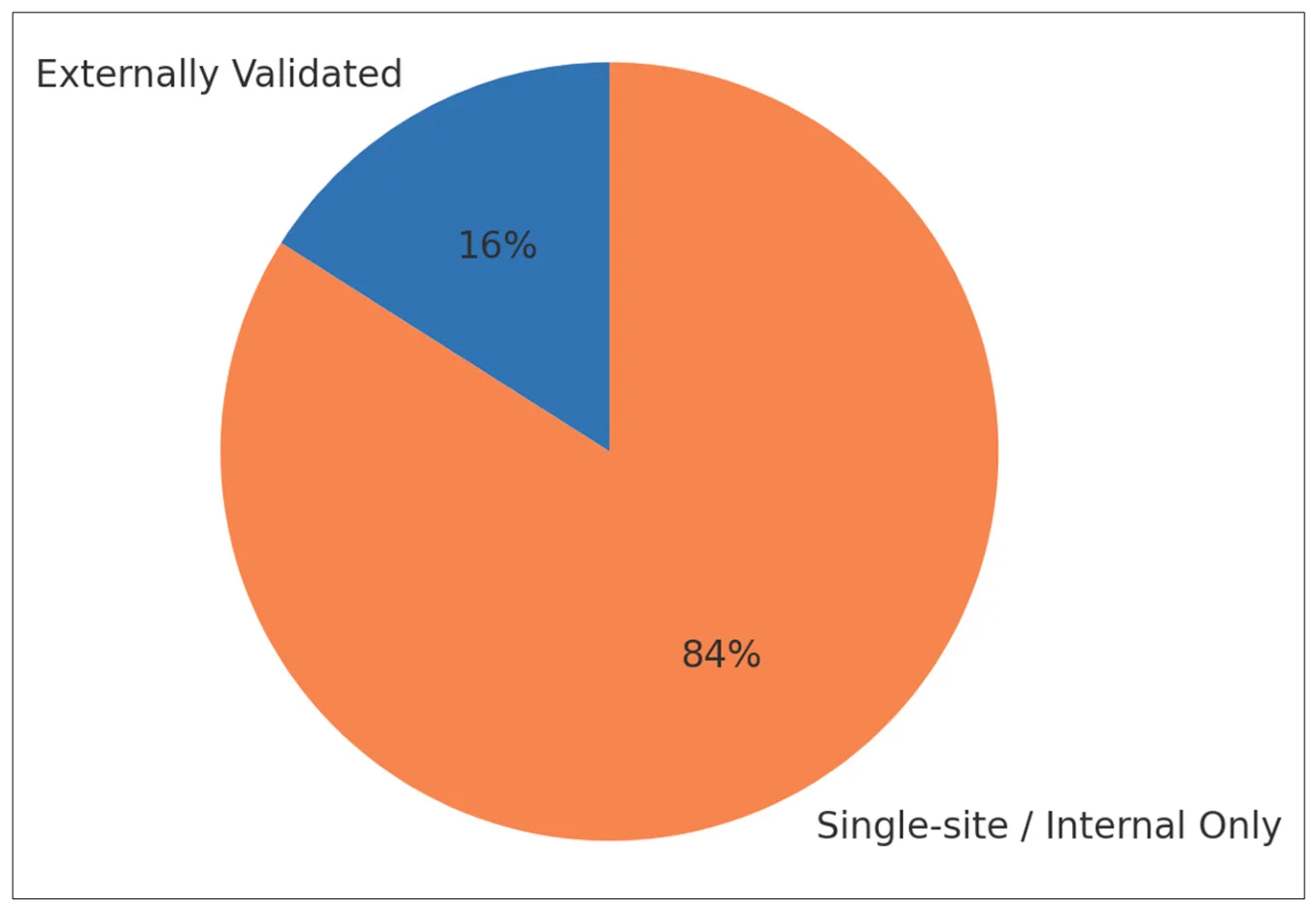
External validation in AI-CDSS studies in the ED.

External validation remains a major gap in AI-CDSSs for the ED. While most studies reported strong internal AUROC values, only a handful tested models beyond their derivation sites. As shown in Table 1 and illustrated in Figure 3, only 16% of reviewed studies included external validation, underscoring the predominance of single-site evaluations in this research domain. Lupei et al. [19] stand out for validating a logistic regression-based CDSS algorithm across 12 hospitals in a single health system, achieving AUROC values between 0.85 and 0.87 on various datasets. Similarly, van de Maat et al. [29] evaluated logistic regression and Classification and Regression Trees (CART) models in two geographically distinct ED cohorts, which are Rotterdam (Netherlands) and Coventry (United Kingdom), thereby enhancing generalizability relative to single-center designs. By contrast, the majority remained single-site evaluations with limited generalizability explicitly acknowledged (Table 1) [16,21,23]. This raises concerns about overfitting to local populations, given that EDs differ widely in patient demographics, resource constraints, and workflow patterns. Without robust multi-center validation, even high-performing AI-CDSS risk limited adoption due to uncertain scalability.

Maintenance and drift are also important issues that were rarely addressed (Table 1). EDs are dynamic environments where disease prevalence, case mix, and practice patterns shift rapidly. Yet, most studies treated models as static, without explicit plans for recalibration or retraining. A notable exception is a study that designed a Bayesian network that automatically adjusted probabilities using physician input and updated patient data [28]. Lupei et al. [19] also acknowledged calibration issues in their COVID-19 model, implying a need for updating over time, though no specific methods were detailed. The absence of explicit maintenance strategies underscores a critical gap because, without continuous recalibration, AI-CDSSs in the ED risks performance degradation and potential patient harm, especially during periods of rapid change such as pandemics.

Transparency and interpretability are also unevenly addressed. Some studies integrated interpretability tools directly into their designs. Higgins et al. [22] used an Explainable Boosting Machine to produce inherently interpretable outputs, while [18,26] applied SHAP values and heatmaps to highlight feature importance in infection and sepsis models. Fujimori et al. [23] presented SHAP bar charts to explain predictions in their aortic dissection tool, and CART approaches [29] naturally provided rule-based transparency. Yet, deep learning studies reported high accuracy without corresponding interpretability, reinforcing the concern that “black-box” predictions limit clinician trust and regulatory approval [17,25]. In the ED, where clinicians must act quickly and justify decisions, lack of transparency directly impedes adoption of AI-CDSSs.

#### 3.1.3. Comparative Appraisal of Methodological Families

The included studies fall into a small number of methodological families, and the distribution itself is informative (Supplementary Table S6). Conventional supervised machine learning accounted for 11 of 23 studies, applied predominantly to structured triage and laboratory data for admission, deterioration and diagnostic prediction. Two studies used deep neural networks for waveform and flow modeling, and one used a transformer-based clinical language model for multi-task prediction from narrative text. One study applied natural language processing to triage notes without specifying a deep architecture. Five studies evaluated proprietary or vendor-supplied models whose architecture was not disclosed, and three contributed perception, governance or pre-implementation evidence without evaluating an algorithm directly. Notably, no included study used reinforcement learning, federated learning, or a generative large language model. Given that these approaches feature prominently in the prospective literature, their complete absence from real-world ED evaluations is itself a finding: the methods most discussed as the future of emergency care AI have not yet been evaluated in deployed ED settings.

A direct comparison of diagnostic accuracy across these families is not supportable from the present evidence base, and we do not attempt one. No included study compared families head-to-head on a shared cohort; target conditions, outcome definitions, case-mix, outcome prevalence and reported metrics differ across studies, and only eight of 20 studies with an evaluable model reported an area under the receiver operating characteristic curve at all (Supplementary Table S5). Apparent differences in reported discrimination between families therefore reflect differences in prediction of tasks at least as much as differences in method. The families can, however, be compared to the dimensions that determine deploy ability. Conventional machine learning offered the most favorable combination for ED use: competitive discrimination on structured data, feature-level interpretability either inherent or readily added, modest training data requirements, and consistent with this, it accounted for the majority of studies that reached prospective or live evaluation. Deep and transformer-based models extended the range of usable input data to waveforms and free text, which is a genuine capability gain, but in this corpus, they were accompanied by no reported interpretability method and by single-site validation only, so their scalability to other EDs remains untested. Proprietary vendor models were the family most often evaluated in multi-site and prospective settings, reflecting commercial deployment, yet they were also the least transparent: architecture, training population and update policy were generally undisclosed, which constrains local validation and complicates the accountability arrangements discussed in Section 3.4. Interpretability did not correlate with methodological simplicity in the way often assumed: nine of 20 studies reported no interpretability provision whatsoever, and these included both conventional and deep models. The practical implication is that methodological choice in ED AI-CDSSs is currently governed less by accuracy than by data modality, transparency requirements, and institutional capacity for validation and maintenance.

### 3.2. Clinical Applications and Use Cases

**Figure 4:**
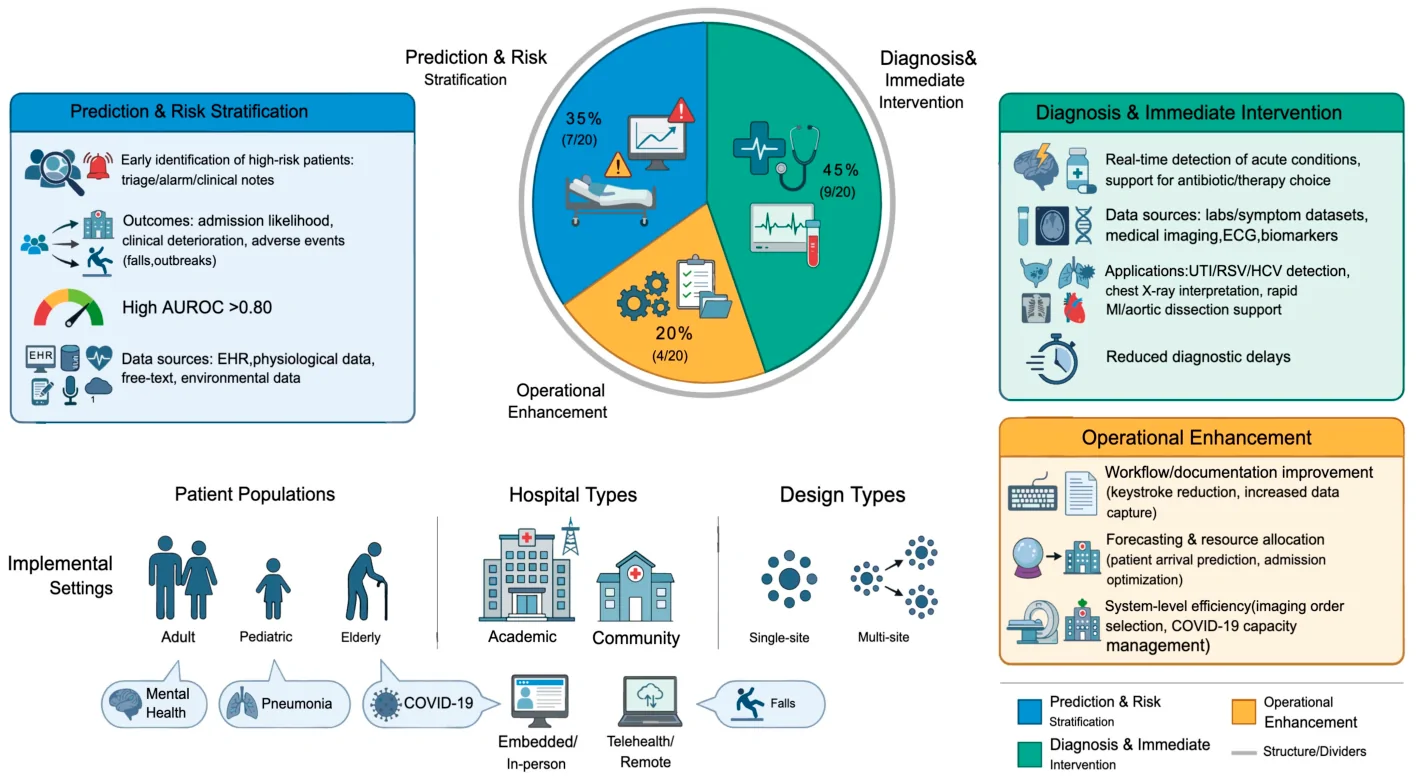
Application categories of clinical implementation of artificial intelligence systems in emergency departments. The graphical layout was created with the assistance of FigureLabs (https://figurelabs.ai) All content, labels, numerical values, and interpretations were reviewed and verified by the authors.

Beyond the technical foundations, the reviewed studies show that AI-CDSSs have been applied to a wide range of clinical and operational tasks in EDs. These applications can be categorized into three major domains: (1) prediction and risk stratification, such as anticipating patient deterioration or admission (35%, seven out of 20 studies); (2) diagnosis and immediate intervention, such as supporting the detection and treatment of acute conditions (45%, nine out of 20 studies); and (3) operational enhancement, such as improving workflow efficiency and documentation (20%, four out of 20 studies) (See Figure 4). This distribution suggests that the literature has focused most heavily on acute clinical decision support, whereas operational enhancement remains comparatively underrepresented despite the central role of workflow pressures in EDs. This variation in application domain is also reflected in the settings in which these tools have been developed and deployed. Implementation settings shape how these applications are deployed across different environments, including adult and pediatric EDs, academic and community hospitals, single-site and multi-site designs, and embedded (in-person) versus telehealth (remote) use.

#### 3.2.1. Prediction and Risk Stratification

Prediction and risk stratification are major application domains for AI-CDSSs in EDs (Figure 4). These tools aim to anticipate downstream outcomes early in the ED encounter, such as the likelihood of hospital admission or clinical deterioration, or adverse events, rather than responding to conditions that are already clinically evident. By identifying higher-risk patients at the point of triage or shortly after arrival, these systems support earlier planning, prioritization, and allocation of clinical resources. The prominence of this application domain suggests that AI-CDSS development in emergency care has been driven in large part by the need to make uncertainty actionable before deterioration or disposition decisions occur.

This anticipatory logic is reflected across studies, although the clinical targets and data inputs differ substantially. Higgins et al. [22] developed ML models to predict mental health inpatient admission using routine triage data and showed that their interpretable model (InterpretML) could support early risk assessment by identifying patients likely to require intensive psychiatric services before disposition decisions were made. Similarly, Akhlaghi et al. [16] implemented a real-time NLP model that uses free-text triage notes to predict hospital admission, finding a negative predictive value (89%) for identifying low-risk patients who could be safely diverted to alternative healthcare pathways, thereby illustrating that early risk estimates can enhance situational awareness and support clinical workflow. Huang et al. [17] extended this predictive capability by leveraging routine physiological data and clinical narratives at triage to predict whether ED patients would require laboratory tests, radiology examinations, medications, or critical interventions during their visits. Using BioClinicalBERT, a pre-trained language model specifically designed for biomedical text, their approach achieved AUROCs ranging from 0.89 to 0.92 across different prediction targets, enabling proactive resource preparation and potentially reducing diagnostic errors. Beyond individual patient risk, Ali et al. [20] used ED and environmental data to forecast infectious disease outbreaks and geographic risk patterns, highlighting the role of AI-driven surveillance for public preparedness and response planning rather than clinical bedside decision-making.

These studies suggest that the value of risk stratification in the ED lies less in prediction as an end than in its ability to change the timing of decisions. Most risk stratification models reported AUROC values above 80%, reinforcing their discriminative potential (Table 1). Barton et al. [33] evaluated the implementation of an algorithm-based clinical decision-support tool that identifies patients aged 65 years and older at high risk for future falls, providing an interruptive alert during the ED discharge process to facilitate referral to a mobility and falls clinics for evidence-based preventive intervention. What links these studies is not merely their predictive orientation, but their shared attempt to shift emergency care from reactive recognition to earlier anticipation of need.

However, while the overall goal of risk prediction is consistent, the data sources, modeling strategies, and clinical endpoints vary substantially across studies. These differences in data inputs and prediction scope suggest that risk stratification is not a uniform application (Table 1). Tylman et al. [28] employed a Bayesian network integrating real-time electrocardiogram (ECG) and vital signs data to detect acute cardiovascular events and mortality, emphasizing sensor-level data acquisition over manual entry of patient records or laboratory results. In contrast, Lupei et al. [19] pursued a generalized logistic regression-based model to forecast severe COVID-19 outcomes across 12 hospitals, prioritizing generalizability and multi-site applicability over disease-specific optimization. These studies show that AI-CDSSs can target different points along the risk spectrum, including imminent physiological deterioration (e.g., cardiovascular events, mortality), disease-specific escalation (e.g., COVID-19 severity), admission likelihood (e.g., mental health, general ED), and specific adverse events (e.g., falls in elderly patients). This heterogeneity is important because it shows that risk stratification in emergency medicine is not a single use case, but a family of related applications that compete between narrow clinical specificity and broader operational relevance. The central challenge, therefore, is not simply to improve predictive performance, but to determine how narrowly or broadly these tools should be designed to remain both clinically useful and transportable across diverse ED populations and settings.

#### 3.2.2. Diagnosis and Immediate Intervention

Another major application domain of AI-CDSSs in emergency medicine is the acceleration of diagnosis and immediate intervention (Table 1). The shared objective across these studies is to reduce diagnostic delays and support timely treatment decisions for acute conditions presenting to the ED. Unlike risk stratification tools that anticipate future events, these systems focus on identifying current pathology requiring urgent therapeutic action. Their prominence in the literature suggests that AI-CDSS have been adopted most readily where the clinical problem is immediate, the consequences of delay are measurable, and the value of earlier recognition can be directly tied to treatment decisions.

This pattern is especially visible in infectious disease applications, where AI is used to shorten recognition time and guide immediate management. Flores et al. [21] developed ML models (RF and neural network) for urinary tract infection diagnosis. These models provide automated risk assessments directly in laboratory reports, influencing antibiotic prescription decisions in real-time at the point of clinical decision-making. Kawamoto et al. [26] focused on respiratory syncytial virus (RSV) infection detection in pediatric patients, achieving a sensitivity of 73.5% and a specificity of 73.9% against rapid antigen testing. Their model helps determine whether patients need to seek medical attention and supports rapid isolation decisions, including in home settings. van de Maat et al. [29] validated seven existing clinical prediction models for childhood pneumonia against a clinical reference standard. They found that three models could moderately discriminate between bacterial and viral infections and could potentially support antibiotic withholding in low-risk children. However, the authors cautioned that threshold selection requires careful consideration of harms and benefits within specific clinical settings. Castro Urda et al. [31] implemented the Intelligen-C strategy for hepatitis C virus (HCV) infection detection. Their approach used AI to automate screening and identify patients with previously undiagnosed infection in the ED, thereby enabling more immediate linkage to care and treatment. These studies suggest that AI contributes most meaningfully to infectious disease settings not by replacing clinician judgment, but by accelerating recognition, supporting triage, and shortening the interval between suspicion and action.

For time-critical cardiovascular emergencies, an AI-CDSSs provides value in supporting rapid diagnosis and intervention. Liu et al. [25] developed an AI-based alarm strategy for acute myocardial infarction (AMI) detection. The system identifies both STEMI and NSTEMI in real-time, automatically alerting physicians via smartphone and facilitating immediate cardiac catheterization laboratory activation. This system significantly reduced door-to-balloon time from 69 to 61 min and ECG-to-cardiac catheterization laboratory activation time from 6.0 to 4.0 min. Pinero de Plaza et al. [12] evaluated the usability and clinical adoption of RAPIDx AI, a decision-support tool designed to assist ED clinicians in assessing suspected cardiac chest pain. The system integrates clinical and biochemical data, including high-sensitivity troponin levels, to generate diagnostic probabilities that distinguish Type 1 myocardial infarction from Type 2 MI and myocardial injury. This distinction is clinically important because interventions such as thrombolysis and coronary angiography are indicated for Type 1 MI but may be harmful in other presentations. Fujimori et al. [23] addressed another life-threatening vascular emergency with their AI-based CDSS for aortic dissection, which generates real-time alerts based on clinical data to reduce the risk of missed diagnoses whose symptoms frequently mimic non-life-threatening presentations. In these studies, the argument for AI-CDSS is strongest when the system does more than classify disease accurately and instead changes the speed or quality of high-stakes intervention.

Complementing these technical evaluations, Rabinovich et al. [32] assessed user satisfaction with an AI system for automated chest X-ray interpretation integrated into ED and radiology workflows. Ease of use was rated highly across clinician groups, but perceptions of diagnostic performance differed between emergency physicians and radiology residents. These findings highlight the importance of distinguishing intended users when designing and implementing AI-based diagnostic tools in clinical settings. However, these diagnostic tools diverge in their input complexity, data requirements, and scope of application. The infection-focused studies varied in their data sources [21,26,29,31], but collectively showed that structured, routinely available clinical data can support real-time or automated diagnostic tools embedded into ED and hospital workflows. In contrast, the cardiovascular applications involved richer, multimodal data integration of physiological monitoring data, ECG signals, and biomarkers reflecting the sensitivity needed for life-threatening conditions [12,23,25]. The radiological AI study [32] represents yet another level of complexity, processing medical images to support visual pattern recognition. This variation is not just technical. It reveals an important tension in the literature. Simpler diagnostic tools are often easier to embed into workflow but may address narrower problems, whereas more sophisticated multimodal systems may offer greater diagnostic precision at the cost of complexity, interpretability, and implementation burden. The central challenge, therefore, is not merely to improve diagnostic accuracy. It is to determine what level of model sophistication can be translated into timely, usable, and trusted support in the time-pressured environment of emergency care.

#### 3.2.3. Improving ED Operations

A third major application of AI-CDSSs in emergency medicine targets operational improvement by optimizing workflows, documentation, and system efficiency rather than focusing only on diagnosis or risk prediction (Figure 4, Table 1). This reflects a pragmatic recognition that the ED functions not only as a site of clinical decision-making but also as a high-pressure operational ecosystem where inefficiencies can cascade into compromised patient care and clinician burnout. By addressing systematic bottlenecks in documentation, resource allocation, and workflow processes, an operational AI-CDSS aims to enhance ED performance without directly substituting clinical judgment. The relative underrepresentation of this domain is notable, because operational inefficiencies are among the most persistent drivers of ED crowding, delay, and clinician burden.

The reviewed studies suggest that operational AI-CDSSs are most useful when they reduce friction in routine processes or improve the alignment between patient flow and institutional capacity. Greenbaum et al. [27] advanced documentation efficiency through an ontology-driven interface that reduced keystroke requirements from 11.6 to 0.6 per chief complaint entry, a 95% improvement, while simultaneously improving structured data capture from 26.2% to 97.2%. This quality-improvement project addressed the burden of data entry on triage nurses and enhanced data quality for downstream analytics, demonstrating how AI can optimize EHR workflows where clinicians reportedly spend 50% of their time on documentation and indirect patient care tasks. Mattay et al. [30] addressed imaging order entry through an AI-assisted predictive text module that guides selection of structured indications based on appropriate use criteria (AUC). Their system reduced unscored imaging orders from 81% to 45%, increasing the proportion of orders eligible for appropriateness scoring and thereby enhancing the potential impact of CDS. While alerts triggered by low AUC scores added only an average of 24 s of provider interaction time, their direct impact on imaging study selection was limited, with providers changing orders based on alert suggestions in only 0.1% of all imaging orders, suggesting that further optimization is needed to translate improved storability into meaningful reductions in unnecessary imaging.

Beyond documentation and ordering workflows, AI-CDSSs have been applied to broader system-level operational challenges. Jiang et al. [34] used deep learning to forecast patient arrival volumes up to 28 days ahead across different triage severity levels, enabling ED managers to optimize resource planning and staff rostering decisions. By predicting demand patterns, their model provided actionable input for operational decision-making aimed at reducing ED overcrowding through proactive scheduling. Hinson et al. [24] developed a clinical decision-support system to optimize COVID-19 hospital admission decisions across a multi-site health system, integrating predictions of clinical deterioration (critical care needs within 24 h, inpatient care needs within 72 h) into a non-interruptive EHR module within the disposition workflow. By focusing on appropriately matching patients to care levels during periods of overwhelming demand, their system addressed healthcare capacity management by embedding clinical risk stratification directly into the disposition workflow, supporting both operational resource allocation and individual patient care decisions. These studies extend the role of AI-CDSS beyond task-level efficiency by showing how predictive tools can also support system-level coordination under conditions of constrained capacity.

The reviewed studies reveal a continuum in how operational enhancement is conceptualized, ranging from narrow workflow optimization to system-level resource management. Greenbaum’s [27] documentation interface and Mattay’s [30] imaging order module represent targeted interventions addressing specific workflow bottlenecks with measurable efficiency gains. In contrast, Jiang’s [34] demand forecasting and Hinson’s [24] disposition optimization operate at a systems level, seeking to harmonize patient flow with institutional capacity constraints. This spectrum is important because it shows that operational AI-CDSSs are not a single class of tools, but a set of interventions that vary in ambition from local workflow support to broader capacity management.

#### 3.2.4. Implementation Settings

AI-CDSS applications in the ED have been tested across a wide spectrum of institutional and patient settings. Most systems were implemented at single sites to establish technical feasibility and workflow integration [16,17,21,27,33]. These implementations allowed for detailed evaluation of clinician acceptance and local workflow integration, though findings may not readily transfer to institutions with different EHR systems or patient populations. The predominance of single-site implementation suggests that much of the literature remains oriented toward proof of feasibility rather than demonstration of scalability.

A smaller group conducted multi-site testing to demonstrate reproducibility across diverse environments. Lupei et al. [19] validated COVID-19 severity prediction across 12 hospitals, prioritizing generalizability over site-specific optimization. van de Maat et al. [29] tested pediatric pneumonia models across two European acute care settings, while Hinson et al. [24], and Pinero de Plaza et al. [12] evaluated cardiac diagnosis and COVID-19 disposition systems across multiple sites, respectively. These studies provide stronger evidence for generalizability but highlight challenges with data harmonization and variable local workflows. This contrast between single-site feasibility and multi-site reproducibility highlights a broader challenge in the literature. AI-CDSSs may perform well under local conditions yet still face substantial barriers when implemented across institutions with different infrastructures and care processes.

Patient populations varied by application. Kawamoto et al. [26] and van de Maat et al. [29] targeted pediatric populations for pneumonia and RSV prediction, while Barton et al. [33] focused on elderly patients (≥65 years) for fall-risk assessment. Most studies focused on general adult ED populations, though Ali et al. [20] extended to population-level infectious disease surveillance. These differences in setting and patient population suggest that AI-CDSSs can be applied across diverse emergency care contexts and adapted to different patient groups. However, the predominance of single-site studies indicates that broader transferability has not yet been established consistently across institutions.

### 3.3. AI-CDSS Implementation Dimensions

**Figure 5:**
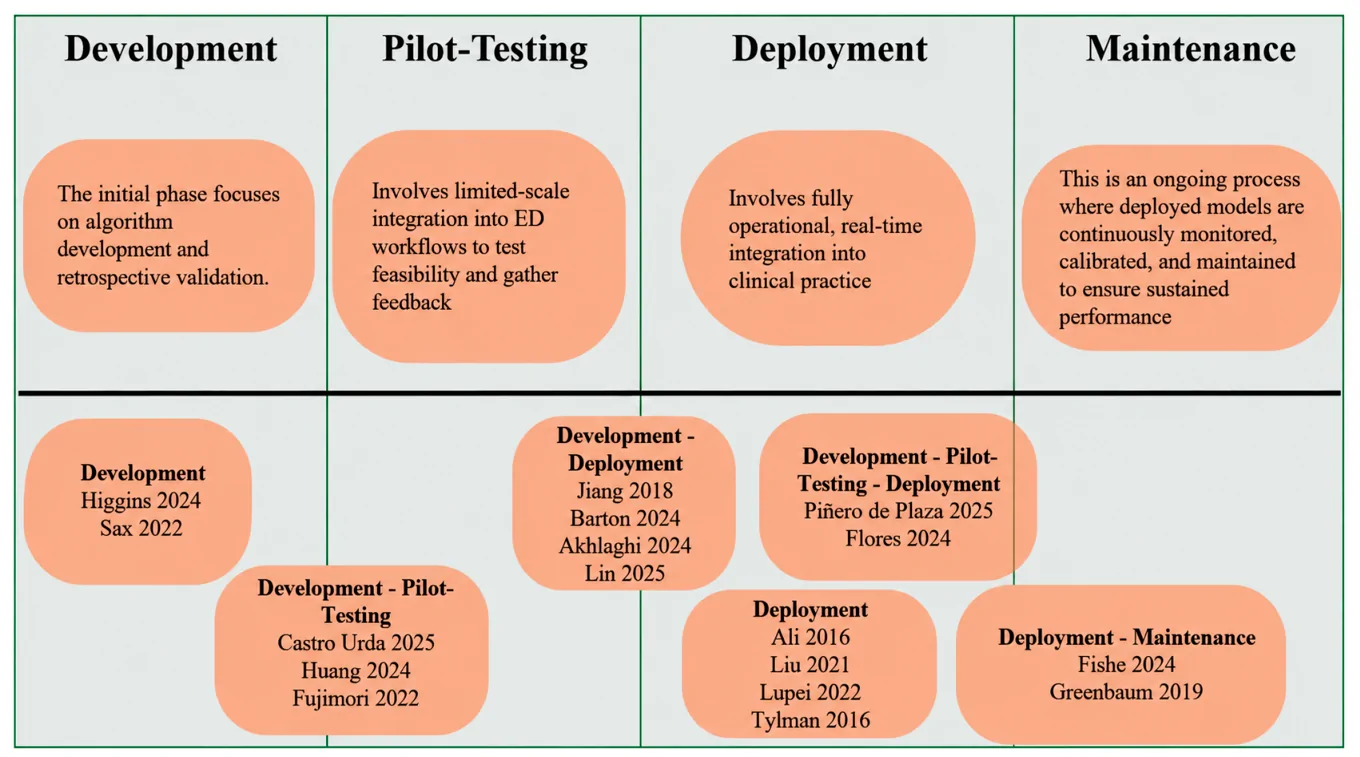
Mapping of reviewed studies across the AI-CDSS implementation lifecycle (development→maintenance) [12,16,17,18,19,20,21,22,23,25,27,28,31,33,34,35,36].

**Figure 6:**
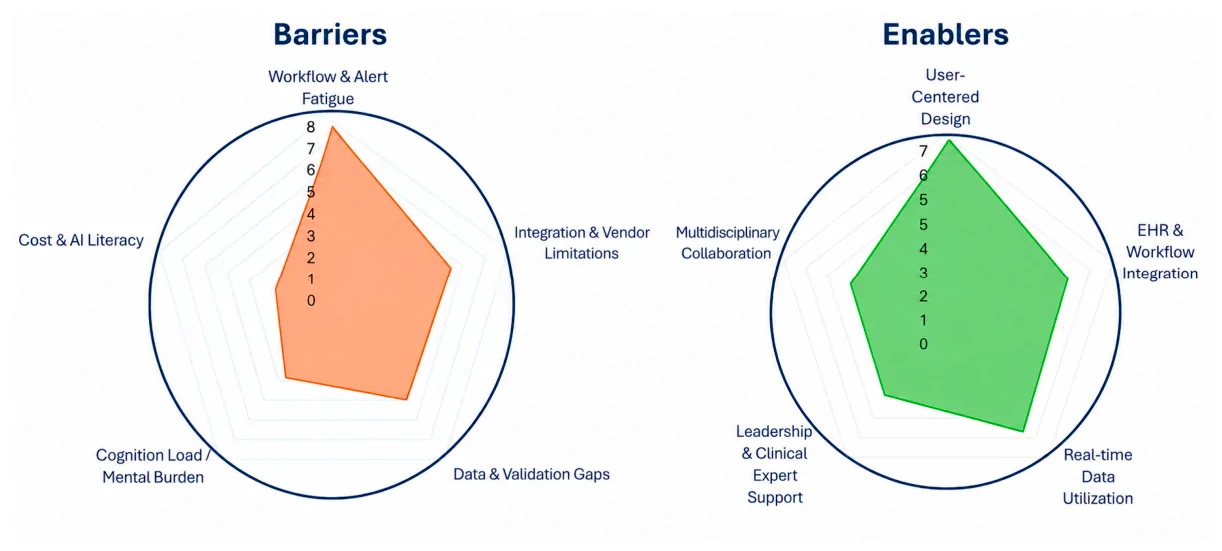
Radar chart visualizing the top barriers and enablers across the implementation dimension.

Although many AI-CDSS studies in EDs reported promising technical performance, implementation outcomes were far more variable and depended heavily on how well these systems fit clinical workflows, user needs, and organizational conditions. While numerous studies reported high technical performance, adoption outcomes varied widely, reflecting the importance of sociotechnical alignment, user experience design, and iterative integration into clinical workflows. As summarized in Table 2 and illustrated in Figure 5 and Figure 6, this variation was evident across development stages, clinical integration, usability, human–AI interaction, and stakeholder engagement. This section examines how AI-CDSSs evolve from conceptual development to embedded clinical tools and why many remain constrained by integration, usability, and workflow barriers despite strong technical results. As illustrated in Figure 6, the challenges and facilitators to implementation are not siloed. Rather, they are embedded across each dimension. Barriers such as workflow fatigue, vendor limitations, and manual burden, and enablers such as user-centered design, real-time data use, and leadership support consistently shape success or failure at each stage of the AI-CDSS lifecycle. Digital-infrastructure characteristics were heterogeneous across studies (Supplementary Table S2). Several AI-CDSS were integrated with EHR/EMR, LIS, RIS, or other clinical systems, but none of the included empirical studies explicitly reported implementation using FHIR or HL7. Data harmonization and real-time source-data acquisition were reported in only a subset of studies, while formal cybersecurity and operational data-governance mechanisms were rarely described. Vendor/platform-related implementation challenges were also inconsistently reported, highlighting remaining barriers to scalable and interoperable deployment.

Eighteen of the 23 included studies provided sufficient implementation-specific information to contribute to this synthesis (Supplementary Table S4).

#### 3.3.1. Implementation Stages

Implementation of AI-CDSSs in EDs spans a progression from algorithm development to pilot testing, full deployment, and ongoing maintenance. Figure 5 maps key studies to their implementation phase or phases and shows that the literature remains concentrated in the earlier stages of maturity rather than in sustained real-world use. Figure 6 complements this pattern by showing that barriers and enablers recur across stages rather than being confined to a single point in the implementation process.

Implementation maturity across reviewed studies ranged from early-stage development to real-world maintenance, but most systems remained in pre-deployment phases. Among the 18 studies, only three (17%) achieved fully operational real-time deployment within ED workflows [20,30]. By contrast, 12 studies (67%) were still in development or pilot testing stages [17,22,23,31,35]. This distribution suggests that the field has produced many technically promising systems, but far fewer have crossed the threshold into routine clinical use.

In the development phase, efforts focused on algorithm creation and retrospective validation, often outside clinical systems. For instance, some studies emphasized technical readiness but lacked live ED integration [20,32]. These studies contributed methodological innovation while also illustrating that technical readiness did not translate easily into clinical use. Progress remained limited by trust and validation gaps, cognitive load and manual burden, and insufficient workflow integration, all of which are reflected in Figure 5. Transitioning into pilot testing, some studies introduced limited-scale deployments to gather usability feedback [17,23,31]. While promising, these pilots encountered issues such as workflow misalignment and alert fatigue, two prominent barrier domains in Figure 5. Pilot testing also suggests that clinical acceptance often hinges more on workflow integration than on technical performance alone.

Only three of 18 studies reached deployment or maintenance, suggesting that implementation maturity is a process of ongoing refinement rather than a single transition from development to use. These implementations benefited from leadership support, clinical expert engagement, and iterative feedback, all of which appear among the major enablers in Figure 6. Similarly, Mattay et al. [30] and Tylman et al. [28] operationalized their tools with clear discharge triggers or academic detailing mechanisms, although broader scalability remained limited. Hybrid pathways also exist, for instance, Flores et al. [21] and Pinero de Plaza et al. [12] progressed through development, pilot testing, and deployment within the same study, demonstrating a more mature implementation cycle that incorporated usability testing, multidisciplinary collaboration, and real-time data utilization. These studies suggest that implementation success depends less on whether an AI-CDSS can be built and more on whether it can be iteratively adapted, integrated into workflow, and sustained beyond initial testing.

#### 3.3.2. The Role of EHR Integration in Adoption Success or Failure

Integration with EHRs or laboratory information systems (LIS) emerged as one of the clearest determinants of whether AI-CDSSs moved toward clinical adoption or stalled despite technical promise. Of the 18 studies, 61% reported at least partial integration into clinical workflows [20,25,30,33]. These studies suggest that adoption improved when AI-CDSSs were embedded within routine clinical systems rather than positioned outside them (See Table 2). AI-CDSSs that achieved full embedding into clinical platforms demonstrated smoother workflows, minimal disruption, and higher clinician uptake. Ali et al. [20] and Barton et al. [33] embedded a geospatial CDSS within Epic across five EDs providing real-time surveillance of infectious disease outbreaks without requiring additional data entry. Akhlaghi et al. [16] and Mattay et al. [30] reported similarly positive outcomes by integrating predictive text modules directly into existing documentation systems. Liu et al. [25] achieved perhaps the most advanced integration, linking a DL system for AMI into the EHR so that 10 s alerts fed directly into existing care pathways. Pinero de Plaza et al. [12] extended this further by embedding RAPIDx AI across 12 Australian EDs in a multi-site Randomized Controlled Trial (RCT), demonstrating that integration at scale is possible when supported by harmonized infrastructure. As summarized in Table 2, these cases suggest that adoption is facilitated when AI-CDSS are embedded within existing clinical systems. This is not because integration alone guarantees acceptance. Rather, integration reduces disruption and aligns decision support with clinicians’ established routines.

By contrast, 39% of reviewed studies lacked full integration into existing clinical systems, and these studies more often encountered adoption challenges even when they demonstrated strong technical performance. Akhlaghi et al. [16] developed an NLP-based triage model that required users to access outputs outside the main workflow, creating friction that limited usability. Greenbaum et al. [27] similarly demonstrated improvements in documentation through an ontology-driven interface, but the lack of integration into the EHR limited generalizability and scalability beyond a single tertiary center. Lupei et al. [19] also revealed the complexities of cross-institutional integration, as their COVID-19 prognostic model required harmonized data pipelines across 12 hospitals, showing that even technically sound models can falter if integration across sites is inconsistent. These studies suggest that adoption declines when AI-CDSSs function as external add-ons rather than embedded aids. Even technically strong models are less likely to succeed when they increase, rather than reduce, clinician workload.

#### 3.3.3. Human–AI Interaction

Human–AI interaction emerged as a central determinant of whether AI-CDSSs supported clinician decision-making or introduced new burdens despite strong technical performance. As summarized in Table 2, about 50% of the reviewed studies explicitly highlighted interaction design, suggesting that the way clinicians engage with AI-CDSS can be as important as model accuracy itself. A central theme across the literature is whether AI should function as a co-pilot that augments human judgment or as an alert-driven mechanism prompting immediate action. Some studies exemplify augmentative interaction models by presenting probabilistic outputs, ranked options, or background surveillance data intended to inform rather than override clinician judgment [13,20,27,28,33]. By contrast, Fujimori et al. [23] and Liu et al. [25] represent more alert-oriented designs in which automated signals were intended to trigger immediate clinical action. These approaches offered speed and decisiveness, but they also introduced concerns about alert fatigue, user trust, and long-term adoption.

This divergence underscores a fundamental trade-off in ED human–AI interaction regarding the degree of autonomy that should be granted to AI in high-stakes, time-sensitive environments. Fujimori et al. [23] illustrate this challenge clearly, as over-alerting and clinician burden were identified as barriers despite the use of explainable outputs. Similarly, Sax et al. [35] reported clinician concern about black-box systems and a preference for minimal disruption, suggesting that clinicians may resist systems that act too autonomously or too opaquely even before live deployment occurs. These findings suggest that effective human–AI interaction requires a careful balance between timeliness, interpretability, and reliability, rather than a simple preference for either augmentation or automation.

Another key dimension of human–AI interaction concerns the degree of explainability and the need for contextual tailoring in implementation. Augmentative approaches often integrate interpretable features that enhance clinician trust. Some studies paired predictive text modules with transparent documentation shortcuts that allowed clinicians to understand and adjust system outputs [30,33], while another study applied SHAP visualizations to clarify feature contributions [23]. Higgins et al. [22] showed that interpretability can sometimes reduce model accuracy, as their Explainable Boosting Machine was more transparent but performed less effectively than black-box models such as XGBoost and Random Forest. Conversely, Akhlaghi et al. [16] developed an NLP-based triage tool that generated predictions with limited explanatory context, which constrained transparency and may have reduced clinician confidence. Liu et al. [25] and Pinero de Plaza et al. [12] implemented technically strong but relatively opaque models that offered limited insight into their reasoning, further constraining trust. Some other studies emphasized that interaction models must be adapted to clinical context [12,35]. In settings involving acute conditions such as AMI detection or sepsis alerts, speed and automation may be prioritized, whereas in documentation or risk stratification tasks, interpretability and shared decision-making are more important. Effective human–AI interaction in emergency medicine therefore depends on achieving a balance between accuracy, timeliness, and transparency while ensuring alignment with the cognitive and operational realities of diverse ED environments.

#### 3.3.4. Usability and User Experience

Across the reviewed studies, formal usability assessment was reported in only 33% of studies, yet usability consistently emerged as a pivotal and often underdeveloped determinant of AI-CDSSs adoption in EDs. As summarized in Table 2, systems that incorporated usability principles early achieved stronger clinician acceptance and measurable workflow improvements in the ED, while those that neglected them often stalled after proof of concept. Greenbaum et al. [27] demonstrated a 95% reduction in keystrokes through an ontology-driven interface embedded within the EHR, showing how usability-centered design can reduce documentation burden while improving structured data capture. Similarly, Pinero de Plaza et al. [12] incorporated adaptive interfaces, embedded feedback, and multi-role usability evaluation in the RAPIDx AI trial, showing that usability must align with clinician experience and workflow context. These studies suggest that usability matters most when design features are embedded within existing workflows rather than added as separate processes. In contrast, many studies introduced technically promising tools but performed little or no structured usability testing [16,22,27,31], leaving questions about system performance under clinical pressure. The absence of standardized evaluations such as System Usability Scale (SUS) scores, heuristic testing, or structured clinician feedback was consistent across much of the literature, even in studies claiming human-centered design principles. This inconsistency reflects a broader trend in which human factors are widely recognized as essential in principle but rarely prioritized in practice, creating a persistent gap between technical innovation and clinical adoption. Sustainable implementation of AI-CDSS in emergency medicine therefore requires that usability be treated as a core design principle from the outset rather than as a secondary consideration after deployment.

#### 3.3.5. Stakeholder Engagement and Implementation Frameworks

Only five of 18 studies (28%) explicitly reported the use of a formal implementation framework, suggesting that structured implementation planning remains uncommon even when stakeholder engagement is recognized as important. Across studies, those that actively engaged frontline clinicians, nurses, and IT staff during design and deployment reported smoother integration, higher trust, and better alignment with workflow realities. Pinero de Plaza et al. [12] provide one of the most structured examples, as their multi-site RAPIDx AI trial explicitly integrated clinician and nurse feedback loops during deployment, tailoring usability features to local contexts. Other studies reported stakeholder input from ED physicians and laboratory staff [21]; nurses, physicians, and quality-improvement teams [33]; physicians and paramedical staff [27]; and multidisciplinary teams [34]. These studies illustrate that engagement is not an optional step but a core determinant of adoption success, ensuring systems are not only technically feasible but also practically usable.

The use of formal implementation-science frameworks remains limited but offers valuable insights where applied. Sax et al. [35] used the Consolidated Framework for Implementation Research (CFIR) during pre-implementation planning, while Fujimori et al. [23] applied both UTAUT and CFIR to evaluate physician trust, usability, and adoption barriers in an aortic dissection tool. They utilized the Unified Theory of Acceptance and Use of Technology (UTAUT) to evaluate physician trust in an AI tool for aortic dissection, demonstrating that perceived usefulness and transparency strongly shaped adoption likelihood. Pinero de Plaza et al. [12] advanced this further by applying the PROLIFERATE_AI framework (a structured approach for evaluating AI implementation in healthcare), explicitly linking user feedback to system modifications during a multi-site trial. These structured approaches contrast sharply with most studies, which either lacked formal frameworks or relied on ad hoc evaluations, limiting the ability to generate generalizable lessons across sites and health systems.

By contrast, studies that neglected stakeholder engagement often struggled to achieve meaningful adoption. Higgins et al. [22] and Huang et al. [17] both developed technically advanced models but reported little or no frontline user involvement, leaving uncertainty about how these tools would fit into real-world practice. Akhlaghi et al. [16] also reported only limited clinician input, and Table 2 identifies integration limits as a continuing barrier despite the tool’s simplicity and high negative predictive value. More broadly, when stakeholder engagement was absent, sparsely reported, or restricted to late-stage feedback, studies offered less evidence that the AI-CDSS had been adapted to the realities of local workflow and decision-making. These cases reinforce that technology-driven development without parallel human-centered engagement risks creating systems that are technically sound but clinically irrelevant.

### 3.4. Organizational, Policy and Ethical Considerations

Successful implementation of AI-CDSSs in EDs depends on more than technical accuracy alone. It also requires organizational readiness, regulatory and legal clarity, clinician trust and accountability, equity and bias mitigation, and supportive policy development. Table 3 summarizes the reviewed studies, which revealed a fragmented but evolving discourse across these domains. Although there is some convergence around ethical commitments and the principle that AI should augment rather than replace clinical judgment, the remaining variation across studies underscores that technical performance alone is insufficient. The future of AI-CDSS adoption in emergency medicine will depend equally on addressing these systemic, organizational, and ethical dimensions.

#### 3.4.1. Regulatory and Legal Frameworks

For this review, legal frameworks refer to enforceable requirements such as HIPAA and the GDPR governing privacy, data use, and related obligations. Regulatory frameworks refer to oversight mechanisms governing the authorization, safety, and lifecycle management of AI-enabled clinical software, such as FDA oversight. Ethical frameworks, such as the Declaration of Helsinki, provide normative principles for responsible research rather than constituting laws or regulations themselves. Although these categories may overlap, they differ in their source, enforceability, and oversight function.

Only four studies explicitly referenced binding legal or regulatory frameworks such as the Health Insurance Portability and Accountability Act (HIPAA) in the United States, the General Data Protection Regulation (GDPR) in the European Union, or emerging national AI regulations [37,38,39,40]. Given that AI-CDSSs are being introduced into high-stakes emergency care environments where accountability and liability are central, this limited regulatory attention exposes a structural vulnerability in how these tools are being brought into clinical workflows, as further detailed in Table 4. By building technically advanced models without embedding them within explicit legal structures, both clinicians and patients are left in a gray zone when adverse outcomes occur, and responsibility is contested.

Only four studies engaged with regulatory mechanisms, and most did so indirectly. Fishe [36] explicitly referenced Food and Drug Administration (FDA) guidance on Software as a Medical Device, underscoring the importance of oversight to ensure safety and accountability. Mattay et al. [30] grounded their predictive text intervention in compliance with the Protecting Access to Medicare Act, highlighting how reimbursement-linked policy can influence adoption trajectories. Others, cited Institutional Review Board (IRB) approvals and adherence to the Declaration of Helsinki, demonstrating compliance with biomedical research ethics but offering little about how these safeguards extend to the unique challenges of AI in real-time emergency workflows [17,25,31]. Higgins et al. [22] similarly reported ethics committee approval and a waiver of informed consent, which addressed human subjects’ oversight but fell short of AI-specific legal frameworks. In contrast, many technically mature systems did not refer at all to regulatory structures, highlighting the immaturity of this domain [19,21,28].

The absence of regulatory alignment is particularly consequential in the ED, where the risks of AI deployment are high. Akhlaghi et al. [16] noted that the lack of national AI healthcare policy frameworks perpetuates uncertainty for both practitioners and developers, while Fishe [36] highlighted that resource-limited EDs face heightened challenges due to limited compliance infrastructure. This gap between technical innovation and slowly evolving legal systems underscores the inadequacy of relying solely on general biomedical ethics approvals to govern AI systems. Progress toward sustainable clinical adoption will depend on embedding AI-CDSSs within adaptive regulatory structures that explicitly address privacy, liability, accountability, and equity within the operational realities of emergency care.

Liability for AI-assisted clinical decisions is governed by two interacting layers: the regulatory pathway through which the software reaches the market, and the civil liability regime that allocates responsibility when a patient is harmed. The three jurisdictions most active in this area differ materially on both, and the differences bear directly on how ED deployments should be governed (Supplementary Table S8).

In the United States, AI-CDSSs are regulated as Software as a Medical Device, reaching the market by 510(k), De Novo or premarket approval, although clinical decision support that satisfies the criteria introduced by Section 3060 of the 21st Century Cures Act may fall outside device regulation where the clinician can independently review the basis for the recommendation—a carve-out whose boundary is precisely where opaque models sit uneasily. Two recent instruments address the specific problem that learning systems change after authorization: the final guidance on Predetermined Change Control Plans (December 2024) allows a manufacturer to specify in advance the modifications it may make without a new submission, and draft guidance on AI-enabled device software functions (January 2025) sets out total product lifecycle and transparency expectations. Civil liability, however, remains a matter of state tort law, with no federal cause of action specific to AI. Malpractice claims lie against the clinician and are decided against the standard of care; product liability claims lie against the manufacturer but are constrained by the learned intermediary doctrine, under which a manufacturer that has adequately informed the prescribing clinician may discharge its duty to the patient. The practical effect is that exposure concentrates on the treating physician.

In the European Union, AI-CDSSs require conformity assessment under the Medical Device Regulation (EU) 2017/745 [42], with most diagnostic and triage applications classified Class IIa or higher and therefore requiring notified body involvement. The AI Act, Regulation (EU) 2024/1689 [43], adds a horizontal layer: AI systems used as safety components of medical devices are high-risk, attracting obligations for risk management, data governance, logging, human oversight, accuracy and robustness, and post-market monitoring. On the liability side the position has recently changed in a way material to this field. The revised Product Liability Directive (EU) 2024/2853 [44] brings standalone software, including AI systems, within the scope of strict product liability, introduces disclosure obligations and rebuttable presumptions of defectiveness and causation where technical complexity makes proof excessively difficult, and must be transposed by Member States by 9 December 2026. The separately proposed AI Liability Directive, which would have harmonized fault-based claims, was withdrawn by the Commission, leaving the revised Directive as the principal harmonized route. The direction of travel is thus to ease the claimant’s evidentiary burden against the developer, in contrast to the United States position.

In China, AI-CDSSs are registered with the National Medical Products Administration under the Medical Device Supervision and Administration Regulations, with technical review conducted against guidance issued through the Center for Medical Device Evaluation; this guidance has developed from review points for deep-learning-assisted decision software issued in 2019 to a consolidated guideline for artificial intelligence medical device software, with emphasis on algorithm traceability, dataset quality and lifecycle change control. Civil liability operates through the Civil Code’s product liability and medical damage provisions, under which the medical institution is ordinarily the primary defendant for treatment-related injury, with a right of recourse against the manufacturer where the product was defective. Institutional responsibility is therefore more prominent than individual clinician liability, while regulatory scrutiny of the software itself is comparatively prescriptive.

For an already licensed tool, the allocation of responsibility between developer and physician turns on a distinction that all three systems recognize but resolve differently: the developer is responsible for the properties of the product, and the clinician for its application to the individual patient. Regulatory clearance or CE marking evidences that the product met the applicable standard at authorization; it does not certify performance in a particular department, on a particular case mix, at a particular point after deployment, and it does not discharge the clinician’s independent duty of care. Three findings of this review bear on where the line should fall in practice. First, performance decay after deployment is real: Hinson et al. [24] documented AUROC declining from 0.91 to 0.85 and 0.89 to 0.80 in prospective use, which is a product property the institution cannot detect without monitoring and cannot remedy without the developer. Second, only three of 20 studies reported any model updating or maintenance provision, so the post-authorization obligations that PCCP mechanisms and AI Act post-market monitoring contemplate are largely unmet in current practice. Third, five studies evaluated proprietary models whose architecture, training population and update policy were undisclosed, which makes it impossible for the deploying institution to assess applicability to its own population and correspondingly difficult to sustain the argument that responsibility has passed to the user. We therefore argue that responsibility for an already licensed AI-CDSS is best treated as shared and contractually explicit rather than resolved by clearance status: developers should be required to disclose training population characteristics, performance by subgroup and update policy, and to support local revalidation; deploying institutions should retain documented override rights, monitor performance on their own population at defined intervals, and record the accountable owner for the system; and clinicians should not be held to a standard that presumes an understanding of the model that current explanation methods cannot supply, as discussed in Section 1. Under-resourced departments are least able to sustain these arrangements, which connects the liability question directly to the distributive equity concerns raised in Section 3.4.3 and by Fishe [36].

#### 3.4.2. Trust, Accountability, and the Role of Clinical Judgment

A consistent theme across the reviewed literature, emphasized in 10 of 18 studies (55%), is that AI-CDSSs should function as decision support rather than decision replacement, with clinicians retaining ultimate accountability for patient care. This principle was evident where AI-generated outputs were designed to supplement rather than replace physician judgment in high-stakes emergency settings [20,25,28]. Mattay et al. [30] implemented structured override documentation for imaging order decisions, while Fishe [36] introduced an “emergency medical exception” feature that allowed full bypass of AI recommendations. Greenbaum et al. [27] similarly noted that nurses retained ultimate responsibility when using ontology-driven documentation support, illustrating how accountability structures were intentionally maintained across different system designs. These studies suggest a growing consensus that AI should serve as a clinical collaborator within established workflows, while final decision-making authority remains with human clinicians.

Flores et al. [21] showed that prioritizing specificity in mental health risk prediction could reduce false positives, but at the cost of sensitivity, raising concern about missed high-risk cases and overconfidence in AI reassurance. In contrast, Barton et al. [33] showed that transparency and workflow alignment can strengthen trust, as their system combined probabilistic output, clinician override, and contextual autocomplete in a way that reduced documentation burden while preserving user control. These studies indicate that trust depends not solely on accuracy, but on a balance of transparency, interpretability, and alignment with clinician values and workflow expectations. Without deliberate design elements that preserve clinical authority, technically strong tools risk rejection. When accountability is maintained and trust is cultivated through explainability and usability, AI-CDSSs can reinforce rather than diminish clinician confidence and decision-making capacity within emergency settings.

#### 3.4.3. Ethical and Equity Considerations

**Figure 7:**
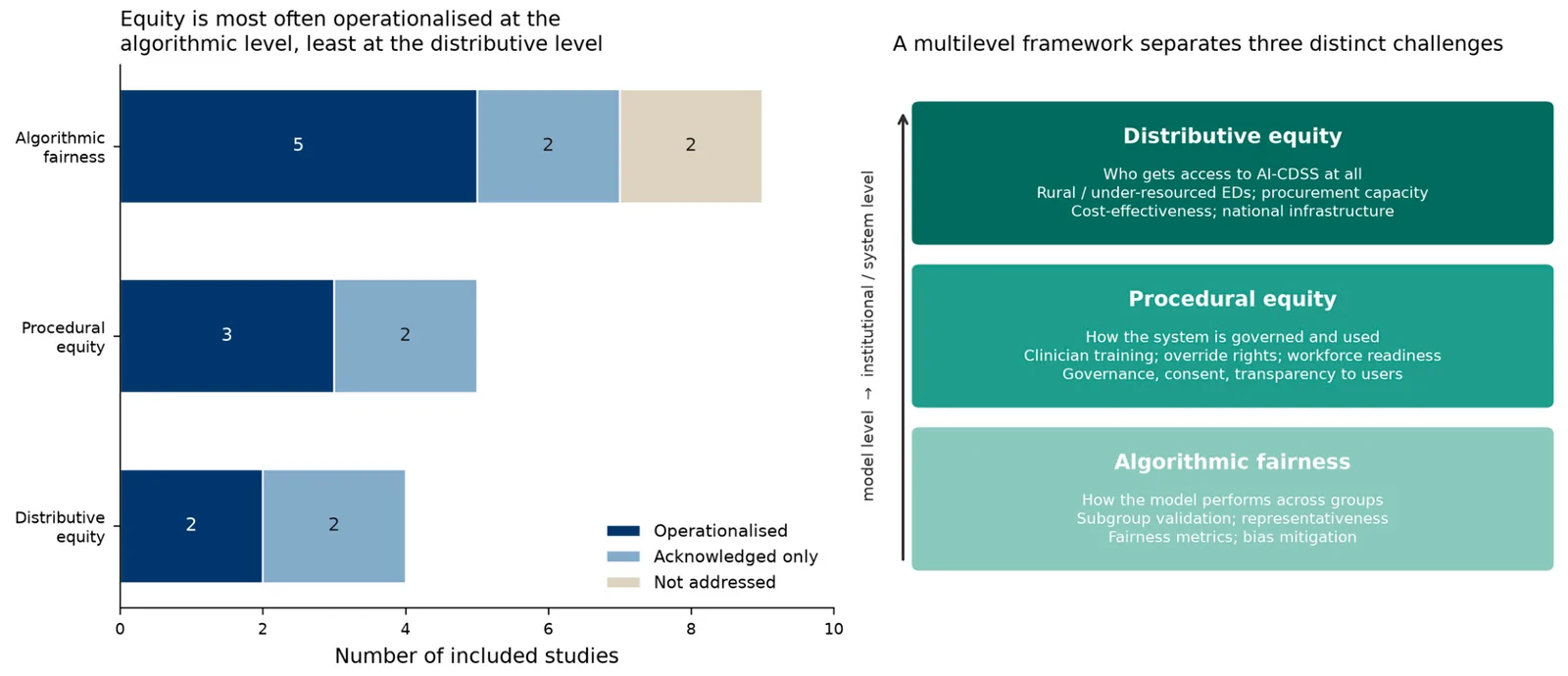
A multilevel framework for equity in ED AI-CDSSs. Left: number of included studies engaging each level, by depth of engagement (operationalized, acknowledged only, not addressed). Right: the three levels and their characteristic concerns. Study-level classifications are given in Supplementary Table S7.

Equity concerns in the literature operate at three distinct levels: algorithmic fairness, procedural equity, and distributive equity. Algorithmic fairness concerns whether a model performs comparably across patient subgroups. Procedural equity concerns whether the processes surrounding the system such as clinician training, override rights, transparency, consent and governance, are designed and applied fairly. Distributive equity concerns which departments and populations have access to these systems. These levels have different causes, remedies and responsible actors; for example, a model may perform comparably across demographic groups while still worsening distributive inequity if it is available only in well-resourced academic centers. Of the 23 included studies, 18 engaged with equity at one or more of these levels. Supplementary Table S7 classifies studies by equity level and depth of engagement, while Supplementary Table S3 provides detailed findings on demographic subgroup performance, socioeconomic factors, racial and ethnic bias, and dataset representativeness. Figure 7 provides a visual summary of these three levels of equity and the depth of engagement across the included studies.

Algorithmic fairness received the greatest attention, although formal evaluation remained limited. Lupei et al. [19] provided the clearest demographic fairness assessment, reporting subgroup performance across gender and racial/ethnic groups using AUROC estimates, 95% confidence intervals, and between-group comparisons. Flores et al. [21] developed sex-stratified prediction models and evaluated their performance by sex. Other studies generally incorporated demographic characteristics or acknowledged potential bias without formally evaluating differential model performance. For example, Tylman et al. [28] incorporated sex and age into Bayesian-network priors but did not conduct subgroup fairness testing, while Higgins et al. [22] discussed potential discrimination in vulnerable mental-health populations without performing subgroup analysis.

Patient-level socioeconomic fairness was particularly underexamined, with no included study systematically comparing model performance across income, insurance, deprivation, or similar socioeconomic strata. Geographic and dataset representativeness were also constrained by the predominance of single-site or single-system datasets. Importantly, no included empirical study reported a formal fairness-specific metric such as demographic parity, equalized odds/equal opportunity, disparate impact, or an equivalent fairness statistic.

Procedural equity was engaged by five studies and is where the review’s implementation focus is most distinctive. Piñero de Plaza et al. [12] documented disparities in clinician confidence and interpretation of model output across experience levels an equity problem located in the workforce rather than the patient population and adapted RAPIDx AI to variation in expertise across 12 EDs. Fujimori et al. [23] deployed SHAP explanations to counter clinician anchoring and tuned alert thresholds to limit alert fatigue, and Barton et al. [33] used academic detailing to address clinician misconceptions in a referral pathway. Sax et al. [35] and Akhlaghi et al. [16] raised bias transparency, privacy, consent and justice but did not describe governance mechanisms to secure them.

Distributive equity was the least developed level, engaged by four studies and operationalized by none directly. Fishe et al. [36] argued that under-resourced and rural EDs lack the financial and legal capacity to implement AI responsibly, raising the prospect of digital marginalization; Lin et al. [18] noted that limited data availability in developing countries permits models trained on non-representative populations to reinforce existing inequities. Castro Urda et al. [31] and Jiang et al. [34] contributed indirectly, the former through cost-effectiveness evidence (€31 per QALY) that bears on affordability in resource-variable settings, the latter through patient-flow modeling intended to support equitable resource allocation. The asymmetry across the three levels is the substantive finding: equity work in ED AI-CDSSs is concentrated where it is technically tractable and largely absent where it requires institutional or policy action.

Operationalizing equity requires specific, auditable practices rather than declarative commitment, and these differ by level. At the algorithmic level, three measures are minimally necessary: pre-specified subgroup analysis, in which discrimination and calibration are reported separately for age, sex, race and ethnicity, language, insurance or deprivation status, and clinically relevant comorbidity groups, with subgroup sample sizes stated; explicit minimum performance standards, whereby an acceptable performance floor is defined in advance for every prespecified subgroup and failure to meet it blocks deployment for that subgroup rather than being reported as a limitation after the fact; and reporting of calibration by subgroup, since a model may discriminate equally well across groups while systematically over- or under-estimating risk in one of them, which is the error mode most likely to alter triage decisions. In the present corpus only one study reported subgroup performance in this manner, and none specified a performance floor in advance. At the procedural level, operationalization means periodic equity audit integrated into model monitoring rather than conducted once at validation—recomputing subgroup performance on the deployed population at defined intervals, monitoring override and alert-acceptance rates by patient subgroup and by clinician grade to detect differential trust, and assigning a named accountable owner for the audit within the institution’s governance structure. Hinson et al. [24], who documented performance decay in prospective use, illustrate why one-off validation is insufficient. At the distributive level, operationalization extends beyond the individual institution: equity-weighted procurement criteria, data-sharing arrangements that allow models to be validated on populations unlike those on which they were trained, and reporting of the case-mix and resource-level of the deploying site so that readers can judge transferability. We recommend that ED AI-CDSS evaluations report, as a minimum, subgroup-stratified performance with prespecified floors, the audit interval and accountable owner, and the demographic composition of both training and deployment populations.

### 3.5. Real-World Impact and Evaluation

**Figure 8:**
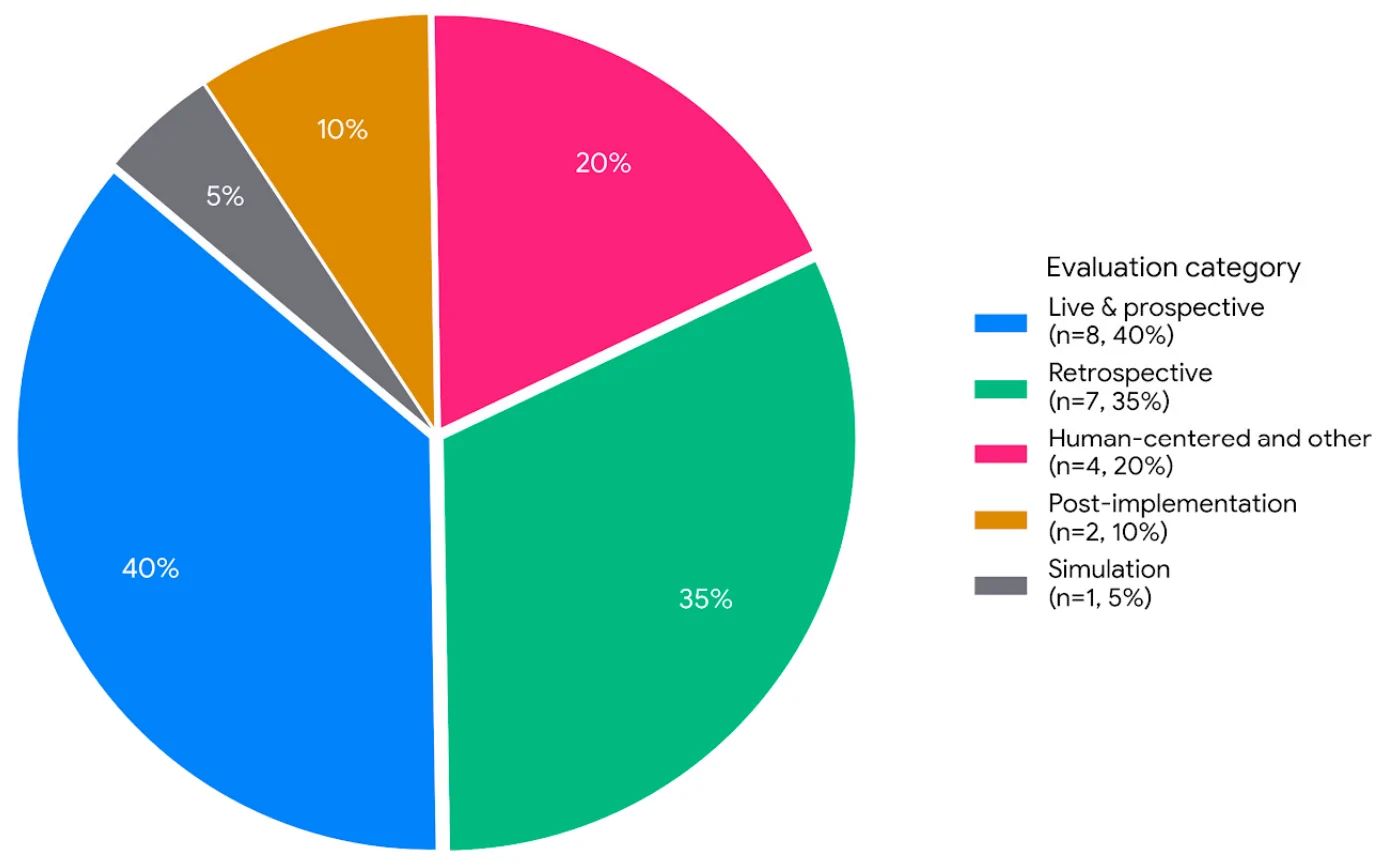
Distribution of studies by implementation stages (categories are not mutually exclusive because studies may report multiple evaluation approaches).

While Section 3.3 examined how AI-CDSSs were implemented in EDs, this section examines how they were evaluated and, more importantly, whether the available evidence is strong enough to support claims of real-world impact. Figure 8 and Table 4 summarize the reviewed studies, illustrating diverse evaluation approaches ranging from retrospective validations and simulation-based assessments to live prospective deployments, post-implementation analyses, and human-centered evaluations, among the 20 studies analyzed in this section, seven (35%) remain confined to retrospective testing, while eight (40%) progress to live or prospective real-world evaluation; the largest single category, reflecting growing but still uneven momentum toward deployment-stage evidence. Simulation-based evaluation remains rare, with only one study (5%) adopting this approach. Post-implementation evaluation is similarly limited, with just two studies (10%) examining AI-CDSS performance and sustainability beyond the point of initial deployment. Human-centered and perception-based evaluations account for a further four studies (20%), collectively foregrounding clinician experience, usability, and acceptance as dimensions that technical validation alone cannot address. These patterns suggest that while methodological sophistication has advanced, a persistent gap remains between algorithm development and robust validation of effectiveness, scalability, and long-term impact, particularly in the domains of workflow integration, clinician adoption, and patient-centered outcomes.

#### 3.5.1. Evidence from Retrospective Evaluations

Seven of 20 studies (35%) relied on retrospective validation, benchmarking algorithmic performance using historical EHR and clinical data, and reporting metrics such as AUROC, sensitivity, and specificity (Table 4). Across these studies, AI models were applied to a wide range of clinical problems, including mental health admission prediction, childhood pneumonia diagnosis, sepsis detection, cardiovascular risk stratification, and infectious disease case recovery. In several cases, models achieved strong discriminative performance, BioClinicalBERT exceeded AUROC 0.90 for predicting laboratory and radiology interventions from triage narratives [17], and an LGBM model using routine CBC + DIFF data demonstrated comparable performance for early sepsis detection [18]. Retrospective cohort analysis also proved valuable for uncovering gaps in routine clinical practice, with AI-assisted record review identifying HCV-positive patients lost to follow-up who had been missed entirely by standard care [29]. However, performance was far from uniform; childhood pneumonia models showed variable cross-site discriminability with only three of seven achieving acceptable thresholds [29], and mental health admission models produced high false negative rates despite reasonable overall metrics [22]. Earlier work, such as the Bayesian network for cardiovascular event prediction, demonstrated promising specificity but remained constrained by very small validation cohorts [28]. Similarly, Lupei et al. [19] achieved strong retrospective AUROCs of 0.87 and 0.82 across temporal and PUI validation cohorts, though generalizability remained limited to a single healthcare system. Collectively, these studies reflect methodological rigor in model development, but consistently fall short of clinical readiness; none formally assessed workflow integration or clinician usability, and retrospective datasets rarely capture the incomplete, time-sensitive, and noisy data that characterize live emergency department environments.

#### 3.5.2. Evaluation in Live and Prospective Settings

Eight of 20 studies (40%) evaluated AI-driven clinical decision-support tools in live or prospective emergency department settings (Table 4). Across these studies, AI was applied to a broad range of clinical problems, from infectious disease surveillance to cardiac emergency detection, imaging documentation, and antimicrobial stewardship. In some cases, deployment yielded striking operational gains, real-time STEMI detection with door-to-balloon time reductions [25], an 8-fold increase in HCV case detection through opportunistic screening [31], and a near-elimination of unstructured imaging orders through predictive text assistance [30]. Bidirectional improvements in antibiotic stewardship, reducing unnecessary treatment in low-risk patients while appropriately escalating care in high-risk ones, were also reported in the context of UTI management [21]. Other studies reported more nuanced findings, where prospective validation across multiple sites revealed expected performance drops from retrospective benchmarks, yet still demonstrated clinically meaningful reductions in mortality among high-risk patients [19,24]. Improvements in workflow efficiency and documentation quality were also reported [27], alongside early-stage syndromic surveillance pilots operating outside high-income settings [20]. Despite this breadth, most studies were conducted at single centers with limited follow-up, and few systematically measured clinician adoption or long-term patient outcomes, leaving the sustained real-world impact of these tools largely uncharacterized.

#### 3.5.3. Simulation Evaluation

Only one of 20 studies (5%) evaluated an AI-CDSS through a simulation-based design before live deployment. Fujimori et al. [23] conducted a pre-deployment mixed-methods evaluation of an XGBoost-based aortic dissection prediction tool across two Japanese emergency departments, using clinical vignettes with 14 physicians alongside UTAUT and CFIR implementation frameworks. While the model demonstrated strong discriminative performance (AUROC 0.901) and user acceptance was generally positive, System Performance and Compatibility emerged as significant barriers, with physicians raising concerns around alert fatigue, diagnostic bias, and workflow disruption, underscoring the value of simulation-based evaluation in surfacing implementation challenges before real-world deployment.

#### 3.5.4. Post-Implementation Evaluation

Two of 20 studies (10%) evaluated AI-CDSS tools after clinical deployment, examining real-world sustainability, usability, and performance in practice rather than at the point of initial implementation (Table 4). Barton et al. [33] used qualitative interviews with 16 ED clinicians to surface five recurring categories of workflow and usability challenges, particularly around alert timing and referral misconceptions, concluding that long-term sustainability requires systems-level redesign alongside targeted education during initial rollout. Akhlaghi et al. [16] took a more quantitative approach, prospectively tracking an NLP-based admission prediction tool and documenting a real-world accuracy decline from 83% to 74% attributable to concept drift and operational complexity, a finding with direct implications for how AI-CDSS tools are monitored and maintained beyond deployment. Together, these studies highlight a critically underexplored dimension of AI evaluation in emergency medicine: that deployment is not an endpoint but a starting condition, and that without continuous monitoring, retraining, and organizational adaptation, even well-validated tools risk gradual performance erosion and clinician disengagement.

#### 3.5.5. Human-Centered and Perception-Based Evaluation

Four of 20 studies (20%) evaluated AI-CDSS tools through pre-implementation assessments, perception surveys, user satisfaction studies, and human-centered evaluations, collectively foregrounding the clinician experience rather than algorithmic performance (Table 4). These studies addressed a dimension of AI adoption that technical validation alone cannot capture how clinicians perceive, accept, and interact with AI tools in the context of their existing workflows and professional identities. Pre-implementation work by [35] identified workflow integration, ML transparency, and outcome feedback as critical facilitators of adoption before any deployment had occurred, while another study found that clinicians held nuanced and sometimes conflicting views about AI-CDS [41], welcoming its potential to standardize care while expressing concern about interpretability and the erosion of clinical gestalt. User satisfaction evaluations revealed further complexity. Rabinovich et al. [32] documented diverging perceptions of output quality between radiology residents and emergency physicians despite high overall ease-of-use ratings, suggesting that acceptance is neither uniform nor predictable across professional roles. At a larger scale, ref. [12] demonstrated across 12 emergency departments that role-based disparities in engagement and comprehension were substantial, with residents and interns facing the greatest implementation barriers, underscoring that technical readiness and clinician readiness are distinct and equally important conditions for successful AI deployment. Together, these studies make a compelling case that human-centered evaluation should be embedded throughout the AI implementation lifecycle, not treated as an afterthought following technical validation.

#### 3.5.6. Evaluation Gap and Real-World Impact

Despite the breadth of evaluation approaches represented across these studies, a persistent imbalance remains. Algorithmic performance continues to dominate the evidence base, while patient-centered outcomes and sustainability are consistently underreported. This gap is particularly important because the ultimate justification for AI-CDSSs is not improved predictive performance alone, but measurable improvement in patient care and outcomes. Several included studies demonstrate that patient-centered evaluation is feasible. Hinson et al. [24] reported reductions in mortality and ICU upgrades during prospective implementation. Liu et al. [25] evaluated one-year Major Adverse Cardiac Events after PPCI, including all-cause mortality, heart failure hospitalization, and nonfatal myocardial infarction, in addition to reporting shorter treatment times; however, the composite MACE outcome did not significantly improve after AI-S implementation. Flores et al. [21] reported improvements in treatment appropriateness, while Castro Urda et al. [31] incorporated Quality-Adjusted Life-Year outcomes into the economic evaluation. These findings show that clinically meaningful patient outcomes can be incorporated into AI-CDSS evaluation, although the available evidence remains too limited to conclude that AI-CDSS consistently improve patient outcomes across ED settings.

Several factors may explain why patient-centered outcomes remain uncommon in AI-CDSSs evaluations. Much of the AI evidence base remains concentrated on model development, validation, and early-stage clinical evaluation, with relatively few studies progressing to prospective assessment of patient benefit [45,46,47]. Patient outcomes are also more difficult to attribute directly to AI-CDSS because clinical effects depend not only on model performance but also on how clinicians interpret and act on model outputs, subsequent treatment decisions, and the surrounding implementation environment [25,47]. In emergency care, clinically meaningful outcomes may occur after the index encounter or outside the implementing institution, making longitudinal follow-up and linkage across data sources important. Linkage of EHR data with administrative, claims, mortality, or clinical-registry data can provide a more complete longitudinal assessment of downstream outcomes [48]. Methodologically, these limitations could be addressed through pragmatic trials embedded within routine clinical workflows [49], cluster-randomized or stepped-wedge designs where individual randomization is impractical [50], and registry-linked evaluations that use routinely collected data to assess longer-term clinical outcomes [48]. Hybrid effectiveness–implementation designs may further enable clinical effectiveness and implementation outcomes to be evaluated simultaneously under real-world conditions [46,51].

Future evaluations should therefore treat patient-centered endpoints as core outcomes alongside algorithmic performance, workflow usability, and implementation measures, and should assess whether technical improvements translate into sustained clinical benefit.

## 4. Future Directions for AI-CDSSs in Emergency Medicine

AI-CDSSs in emergency medicine have reached a point at which further progress will depend less on demonstrating technical promise and more on overcoming the barriers that continue to limit durable clinical adoption. Although research has shown strong progress in prediction, diagnosis, and workflow optimization, most systems remain fragmented and limited to pilot or retrospective phases. To translate these advances into lasting clinical value, future work must go beyond algorithmic accuracy and address the organizational, ethical, and policy constraints that limit adoption. The next phase of research therefore requires a coordinated agenda that strengthens technical reliability, embeds human-centered evaluation, and aligns development with transparent governance. Only by integrating innovation, usability, and accountability can AI-CDSSs evolve from research prototypes into sustainable tools that improve the quality and timeliness of emergency care.

### 4.1. Technical and Clinical Innovation

Future development must focus on improving both the technical sophistication and clinical relevance of AI-CDSSs. Current models perform well in retrospective validations but often fail to maintain reliability under real-world conditions where data are incomplete, noisy, and rapidly changing. Future research should prioritize multimodal data integration that combines structured EHR data, continuous vital signs, laboratory and imaging data, and clinical narratives [19,22]. Explainability mechanisms such as SHAP visualizations [23] and recalibration methods [28] will be essential for sustaining performance and clinician trust. Future systems should also expand beyond narrow diagnostic outputs to support broader clinical pathways that align with actual emergency workflows [27]. Such systems must undergo multicenter, prospective validation to ensure generalizability across diverse populations and institutional contexts [17,29]. The central challenge, therefore, is not only to build more sophisticated models but to develop systems that can adapt to local data and clinical context while preserving predictive accuracy and practical usefulness.

### 4.2. Implementation Science and Human-Centered Design

Technical success alone is insufficient without structured implementation and user-centered evaluation. Future studies should embed implementation-science frameworks such as CFIR, UTAUT, or PROLIFERATE_AI [12,35]. These frameworks provide a foundation for evaluating contextual factors, adoption barriers, and long-term sustainability. Human-centered design should guide all stages of development, ensuring that AI-CDSSs complement existing workflows rather than disrupt them. Evidence from [23,33] shows that clinician engagement, usability testing, and transparent reasoning are critical to trust and sustained use. Co-design with end users, simulation-based usability trials, and iterative feedback loops can bridge the gap between technical potential and clinical integration. Other studies further suggest that when design and workflow alignment are prioritized, clinician acceptance and operational fit improve [16,25]. Integrating implementation science with human-centered design therefore offers one of the clearest paths toward meaningful and scalable adoption of AI-CDSS, because it addresses not only whether a system works, but whether it can be trusted, used, and sustained in routine emergency care.

### 4.3. Governance, Policy, and Equity

Progress in AI-CDSSs will depend on governance systems that evolve alongside technological advances and ensure accountability, transparency, and equity. Several included studies emphasized that the lack of AI-specific regulation creates uncertainty around liability, privacy, and institutional responsibility [31,36]. Most studies referenced only general ethical approvals such as IRB clearance or adherence to the Declaration of Helsinki, which remain insufficient for dynamic, real-time AI systems. Future governance frameworks should therefore move beyond one-time certification and adopt adaptive oversight that includes conditional approval, iterative evaluation, and continuous post-deployment monitoring. Transparency standards, explainability requirements, and regular reporting of model updates are essential to maintain clinician trust and public accountability. Governance must also be designed to promote equity by supporting under-resourced EDs that lack the technical and financial capacity to sustain AI deployment independently. Public investment, interoperability standards, and national oversight can reduce these disparities and ensure consistent access to safe and effective AI-CDSS [17,36]. Bias-mitigation practices, such as subgroup validation and equity audits [23], should likewise move from conceptual discussion toward routine operational use. At the policy level, balance will be essential: excessive regulation may constrain innovation, whereas insufficient oversight may perpetuate inconsistency, liability concerns, and inequity. National guidance on transparency, privacy, reimbursement, and accountability will therefore be important for sustainable and trustworthy AI-CDSS integration across emergency care systems [16,30].

Recent health-technology policy briefs illustrate how synthesized evidence can be translated into practical, content-sensitive policy options [52,53,54]. Applied to emergency care, our findings suggest that health systems should require local or multi-site validation before AI-CDSS deployment, particularly when models are transferred across settings or patient populations. Decisions about adoption should consider expected clinical benefit alongside feasibility, resource requirements, and equity, consistent with the GRADE Evidence-to-Decision framework [55]. Interoperable and secure data-governance standards are also needed to support reliable integration with existing clinical systems, while post-deployment monitoring should extend beyond technical performance to include model drift, safety, and equity. Procurement and reimbursement decisions should similarly consider evidence of clinical benefit and equitable performance rather than predictive accuracy alone. Responsibility for these activities is shared: regulators can establish minimum safety and reporting requirements, health systems can oversee local validation and ongoing monitoring, developers can provide greater transparency regarding training data and model updates, and payers and procurement bodies can reinforce these expectations through contracting and reimbursement. Importantly, policy oversight should continue after deployment. The NASSS framework highlights the importance of considering how technologies adapt, scale, and remain sustainable within changing organizational and clinical environments [56]. Evidence generated during routine use should therefore inform periodic reassessment and policy adaptation as AI-CDSS and emergency-care settings evolve.

Governance will also need to specify what equitable deployment requires in operational terms rather than in principle. On the evidence of this review, three requirements are minimal. Evaluations of ED AI-CDSSs should report subgroup-stratified discrimination and calibration against performance floors defined in advance, rather than reporting subgroup disparity as a post hoc limitation. Deploying institutions should conduct periodic equity audit as part of routine model monitoring, with a named accountable owner and surveillance of override and alert-acceptance rates by patient subgroup and clinician grade. Procurement and funding mechanisms should incorporate equity-weighted criteria and support data-sharing arrangements that allow models to be validated in departments unlike those in which they were developed, since the departments least able to fund local validation are those where unvalidated deployment carries the greatest risk.

### 4.4. Data and Algorithmic Advancement

The next generation of AI-CDSSs should prioritize not only stronger algorithms, but also more diverse, higher-quality, and more adaptable data foundations. Current tools rely heavily on structured data and often overlook multimodal signals that capture the full complexity of emergency care. Combining laboratory results, imaging, monitoring data, and free-text narratives can substantially improve predictive precision and timeliness. To ensure reliability, preprocessing pipelines must manage missingness, data noise, and variable documentation quality without introducing additional bias. Algorithms should evolve from static models into adaptive systems capable of recalibrating in response to shifting patient populations or clinical conditions. Techniques such as federated learning and continual updating can maintain performance without requiring centralized data aggregation. Hybrid modeling approaches that balance interpretability with predictive performance, such as interpretable boosting machines or ontology-based classifiers, may further strengthen clinician trust while preserving practical usefulness. Embedding transparent feature importance metrics, SHAP explanations, and causal inference tools can strengthen understanding and accountability. Finally, algorithmic fairness must be operationalized through balanced sampling, subgroup validation, drift monitoring, and equity-driven optimization to ensure that predictive gains do not come at the expense of vulnerable populations.

### 4.5. Sociotechnical Integration and Sustainability

The sustainability of AI-CDSSs will depend on how effectively they are integrated into the sociotechnical realities of emergency care. Human factors such as trust, cognitive load, and workflow compatibility are as decisive as algorithmic precision. Future systems should provide layered explainability, with concise summaries for routine use and more detailed justification when needed. They must reduce rather than increase documentation burden and cognitive workload, drawing lessons from predictive text tools that improved efficiency [33]. Integration must also reflect the collaborative nature of emergency care. Systems should tailor information presentation for different users, including concise triage indicators for nurses, detailed reasoning displays for physicians, and high-level operational dashboards for administrators. Continuous monitoring of user interaction and adaptive interface design can help sustain long-term engagement. Treating human and organizational dimensions as core design elements, rather than as afterthoughts, will be essential if AI-CDSS are to function as trusted collaborators in emergency care rather than as short-lived technical experiments.

## 5. Limitations

This review has several limitations that should be considered when interpreting its findings. First, only three electronic databases (PubMed, Scopus, and Embase) were searched; additional sources such as CINAHL, IEEE Xplore, or ACM Digital Library may contain relevant literature not captured here. Second, we did not conduct a structured quality appraisal or risk-of-bias assessment of the included studies. Consequently, the certainty of the evidence underlying the synthesis could not be formally evaluated, and the findings should be interpreted with appropriate caution, particularly given the predominance of single-site evaluations without external validation. Third, gray literature, including conference proceedings, government reports, and non-peer-reviewed implementation reports, was not systematically searched, potentially omitting evidence on real-world deployments that are less likely to be formally published. Finally, publication bias may have affected the evidence base, as studies reporting positive findings or strong model performance may be more likely to be published than negative results, failed implementations, or discontinued deployments.

Further limitations concern the synthesis of model performance. Reported metrics were heterogeneous, and only eight of 20 studies with an evaluable model reported AUROC. Consequently, performance could not be meaningfully pooled or ranked across studies and is presented as originally reported in Supplementary Table S5.

## 6. Conclusions

This systematic review of 23 studies highlights both the progress and persistent gaps in the development and implementation of AI-CDSSs in EDs. While many models, often based on ensemble learning, deep neural networks, or hybrid approaches, achieved high discriminative performance, the majority remain confined to retrospective validations, limiting their ability to reflect the noisy, incomplete, and dynamic nature of real-time emergency medicine. Prospective and pilot deployments demonstrated the importance of iterative testing and clinician feedback, yet such approaches were inconsistently applied, and long-term evaluations of patient-centered outcomes were rare. Across studies, recalibration and drift detection mechanisms were seldom described, raising concerns about sustainability in rapidly evolving ED environments. Systems that progressed toward full operational maturity shared common enablers, including integration into EHRs, workflow-sensitive design, and clinician engagement, whereas models that remained siloed or poorly aligned with practice often stalled despite strong technical performance. These findings reinforce that algorithmic excellence alone does not ensure clinical adoption. Usability, institutional readiness, and alignment with frontline decision-making are equally critical.

The organizational, ethical, and regulatory gaps that continue to constrain adoption are equally important. Trust and accountability consistently emerged as decisive factors, with clinicians favoring systems that supported rather than supplanted their judgment, and override mechanisms serving as essential safeguards. Yet explainability was explicitly addressed in less than a third of studies, regulatory compliance with frameworks such as HIPAA, GDPR, or FDA guidance was largely absent, and equity was acknowledged more often in principle than embedded in practice. Few systems incorporated systematic bias mitigation, raising the risk of exacerbating disparities across populations and institutions. Encouragingly, a subset of studies demonstrated the value of stakeholder co-design, structured implementation frameworks, and leadership support in building clinician confidence and facilitating uptake. Looking forward, the sustainable integration of AI-CDSSs into emergency care will require moving beyond retrospective accuracy benchmarks toward multi-site, prospective validation. It will also require embedding human-centered design and explainable AI into development, establishing adaptive governance models that balance innovation with accountability, and addressing equity at both the patient and institutional level. AI-CDSS should ultimately be understood not as static tools but as evolving collaborators, designed to augment, rather than replace, human expertise in the high-stakes environment of the ED.

## Figures and Tables

**Table 2:** Characteristics of AI-CDSS implementation studies in emergency departments.

Study	EHR/EMR Integration	Human–AI Interaction Model	Usability/UX	Stakeholder Engagement	Implementation Framework	Key Barriers	Key Enablers
Higgins et al. [22]	n/s	n/s	Not assessed	n/s	-	Limited reporting	Practical recommendations
Greenbaum et al. [27]	Standalone; vital signs monitor	Risk assessment support	Simplified setup	Physicians, paramedical staff	-	Data quality, signal noise	Real-time acquisition; existing infra
Lin et al. [18]	Visual analytics; no centralized EMR	Interactive visualizations	Large displays	Epidemiologists, public health	-	Data scarcity	Real-time data; early detection
Sax et al. [35]	EHR integration planned	ML-based CDS	Prefers minimal disruption	Direct physician input	CFIR	Black-box hesitancy	Training; education; timely alerts
Huang et al. [17]	n/s	n/s	n/s	n/s	-	Single-site; binary limits	Proof of concept shown
Castro Urda et al. [31]	n/s	Patient-entered symptoms	Electronic templates	Patients, pediatric physicians	-	Single-site; internal validation only	Reduces invasiveness; streamlines diagnosis
Fujimori et al. [23]	Numeric + text (NLP)	Alerts w/clinician input	Non-disruptive; intuitive UI	14 physicians (2 hospitals)	UTAUT, CFIR	Alert fatigue; overreliance	Explainable outputs; local adaptation
Flores et al. [21]	LIS-integrated comments	RF/NN hybrid; workflow-embedded	Integrated workflow	ED physicians, lab staff	-	Deployment reproducibility	User involvement; iterative testing
Pinero de Plaza et al. [12]	Full EMR integration	Multi-role feedback; adaptive UI	Tiered feedback; novice issues	Clinicians, IT, patients	PROLIFERATE_AI	Manual data; overload	Co-design; iterative feedback
Mattay et al. [30]	Epic EMR + CareSelect	Predictive text mapping	Workflow friction; alert fatigue	n/s	-	Free-text reliance	Improved functionality; minimal burden
Jiang et al. [34]	EMR with alerts	Complementary screening	Not tested	Multidisciplinary team	-	Follow-up, adherence	Automated screening; team support
Ali et al. [20]	Epic EHR	Mixed human–AI risk estimates	Non-interruptive CDS	5 EDs, academic system	-	Single-system limits	Full embedding; interpretable outputs
Liu et al. [25]	Real-time ECG upload	Automated alerts; human confirmation	Rapid (<10 s)	n/s	-	Single-site; complexity	Real-time integration; expert-level perf
Tylman et al. [28]	12-hospital EHR	Augmentative risk scoring	0–100 scale	ED physicians, experts	-	Interpretation dependency	Readily available data; optimized resources
Barton et al. [33]	Fully integrated	Probabilistic output + override	95% fewer keystrokes; faster docs	Nurses, physicians, QI team	-	Staff turnover; data habits	Contextual autocomplete; ontology design
Akhlaghi et al. [16]	Power BI; partial EMR link	n/s	Simple; low cognitive load	Limited clinician input	-	Concept drift; integration limits	Cloud-based; high NPV
Lupei et al. [19]	Fully integrated; EHR sidebar across 12 hospitals	Risk score supporting shared disposition decisions	n/s	System leadership + multidisciplinary team + frontline providers	-	Generalizability; calibration limitations; external validation needed	Standardized EHR mapping; multi-site deployment; frontline engagement; interpretable risk score
Fishe, [36]	Vendor-dependent	n/s	n/s	n/s	-	Cost; vendor lock; low literacy	Policy support; ED-specific focus

n/s = not specified.

**Table 3:** Organizational, policy, and ethical considerations in AI-CDSS implementation.

Study	Organizational Considerations	Regulatory/Legal Framework	Trust & Accountability	Ethical/Equity Considerations	Bias-Mitigation Strategies
Higgins et al. [22]	Local health district collaboration; site-specific approval	Ethics committee approval; consent waiver	Shared accountability (govt, researchers, clinicians)	Bias concerns for vulnerable groups	Bias acknowledged; limited mitigation detail
Flores et al. [21]	Integrated with lab, ED physicians, existing info systems	n/s	Clinicians retain final authority	Limited ethical discussion	None detailed
Tylman et al. [28]	Standalone device with vitals monitoring	n/s	Medical personnel retain accountability	Minimal discussion	BN root probabilities by admission proportion
Greenbaum et al. [27]	Standardized workflow, quality metrics	n/s	Nurses retain override capability	Burnout reduction focus	Single-center model limits generalizability
Castro Urda et al. [31]	Streamlined diagnostics; reduced invasive testing	IRB & Helsinki compliance	Rapid testing within defined thresholds	Child-centered, reduced discomfort	No explicit bias mitigation
Jiang et al. [34]	Multidisciplinary coordination; hospital management support	Legal & bioethical clearance	Maintains clinical judgment	Quality-of-life, prognosis emphasis	Complementary to traditional screening
Hinson et al. [24]	Admin data leveraged for mgmt planning	n/s	Supports A&ED managers	n/s	Calendar/meteorological data included
Ali et al. [20]	ED resource optimization	n/s	EHR-embedded; physician final decision	Focus on capacity & workflow	Demographic & clinical features included
Lin et al. [18]	Public health data gap addressed	n/s	Multi-level decision support	Equity in resource allocation	Dynamic syndromic classification
Sax et al. [35]	Workflow planning; clinician preferences	n/s	Conservative bias preference; override	Bias transparency focus	Transparency emphasis; no formal plan
Akhlaghi et al. [16]	Privacy, risk, and safety concerns noted	Lack of overarching AI policy	n/s	Privacy, consent, justice emphasized	n/s
Pinero de Plaza et al. [12]	Infrastructure & training investment	n/s	Experience-level trust variation	Inclusive design for diverse users	Adaptive design for equity
Fishe, [36]	Cost & vendor support needs	FDA SaMD regulations cited	n/s	Resource inequity concern	Advocates proactive inclusive design
Mattay et al. [30]	Alert fatigue; integration challenges	PAMA compliance	Emergency exception for CDS bypass	n/s	n/s
Huang et al. [17]	Research ethics approval; Helsinki compliance	n/s	n/s	n/s	Statistical sampling, cross-validation
Fujimori et al. [23]	Physician alert workflow	n/s	n/s	n/s	SHAP-based transparency; alert thresholds
Liu et al. [25]	IRB approval; Helsinki compliance	n/s	Physicians retain diagnostic authority	n/s	n/s

Abbreviations: ED = emergency department; EHR = Electronic Health Record; IRB = Institutional Review Board; BN = Bayesian network; A&ED = accident & emergency department; FDA = Food and Drug Administration; SaMD = Software as a Medical Device; PAMA = Protecting Access to Medicare Act; SHAP = Shapley Additive Explanations; n/s = not specified.

**Table 4:** Real-world evaluation of AI-CDSSs in emergency departments.

Study	Evaluation Stage	Evaluation Approach & Metrics	Workflow/Usability Outcomes	Patient-Centered Outcomes	Scalability/Sustainability Insights
Higgins et al. [22]	Retrospective validation	Six ML models; CatBoost best (MCC 0.20); InterpretML most interpretable	InterpretML explanations; no workflow testing	Reduces false negatives; improves safety	Single site; limited generalizability; external validation needed
van de Maat et al. [29]	Retrospective (multi-site)	Seven models; 3 met ORC ≥ 0.55	Not assessed	Not assessed	Variable performance across sites; biomarker dependent
Lin et al. [18]	Retrospective validation	LGBM AUROC 0.90; Sens 83%; Spec 85%	Web AI-CDSS; training provided	Not assessed	Single-site; CBC-specific tech required
Castro Urda et al. [31]	Retrospective + prospective	8× serology increase; €31/QALY cost-effective	Visits ↓>50%	+2.5 QALYs per patient	Single site; regional scalability potential
Huang et al. [17]	Retrospective validation	BioClinicalBERT AUROC > 0.90 (multi-task)	Proof of concept; no workflow testing	Not assessed	Needs multi-site validation
Fujimori et al. [23]	Pre-deployment simulation	XGBoost AUROC 0.901; SHAP explanations, mixed-method UTAUT quantitative/CFIR qualitative	Positive UX; reduced alert fatigue	Not reported	Single disease; simulated testing only
Barton et al. [33]	Post-implementation	Qualitative interviews (n = 16 ED clinicians)	Workflow challenges; misconceptions in referral process	Not assessed	Requires system-level redesign; education critical
Mattay et al. [30]	Pilot deployment	Predictive text for imaging	Unscored orders ↓81%→45%; +24 s interaction	Not reported	Single site; early-stage deployment
Pinero de Plaza et al. [12]	Human-centered eval	PROLIFERATE_AI; mixed methods (12 EDs)	Role-specific barriers; registrar adoption highest	Not reported	12 diverse EDs; needs tailored implementation
Flores et al. [21]	Full clinical implementation	AUC 0.81–0.88; 3-stage real-world evaluation	UTI risk alerts reduced unnecessary cultures; improved prescribing	Correctly treated ↑372→491; inappropriate Rx ↓	Single site; low-cost replication possible
Hinson et al. [24]	Prospective validation	AUROC decline (0.91→0.85 CCU; 0.89→0.80 inpatient)	Real-time, embedded CDS (1–10 risk); stable deployment	Mortality ↓6.7→2.9%; ICU upgrades ↓11.4→8.5%	Multi-site; pandemic-stable; ongoing training needed
Liu et al. [25]	Prospective validation	F1 0.93 (STEMI); alerts <10 s post-ECG	Integrated ECG & alerts; door-to-balloon ↓69→61 min	1-yr MACE unchanged	Sustained 4 mo; needs RCTs for scalability
Tylman et al. [28]	Retrospective validation	BN + ECG segmentation; Sens 53–68%; Spec 90–100%	Fully automated, no manual data	MI/SCA focus; STEMI 100%, NSTEMI 54%	n = 29; multi-monitor validation needed
Ali et al. [20]	Pilot implementation	Syndromic classification > 95% acc.; dengue forecast NRMSE 0.28–0.29	Real-time outbreak alerts; GIS visualization	Early outbreak detection	Pilot only; reproducibility validated; limited scale
Greenbaum et al. [27]	Pre→post (12 m baseline, 32 m dev, 16 m eval)	HaPPy ontology + ML UI; n = 279,231 encounters	Keystrokes ↓95%; structured data ↑26→97%	Better data quality, coordination	Single center; site-specific ontology limits scaling
Akhlaghi et al. [16]	Post-implementation	NLP accuracy ↓83→74%; Sens 73%; Spec 74%	EMR + Power BI integration; no workflow outcomes	Not reported	First real-time AI in Aus ED; retraining needed
Lupei et al. [19]	Retrospective→prospective	AUROC 0.82–0.87; ICU/vent/death outcomes	EHR sidebar risk scores	Fair by gender/race; severe COVID 0% vs. 33%	12-hospital network; long-term effect TBD
Rabinovich et al. [32]	User satisfaction	Clinician surveys on AI X-ray analysis	User acceptance focus	Not assessed	Acceptance-focused; lacks clinical outcome data
Sax et al. [35]	Pre-implementation	Mixed methods (8 intvw, 67 survey); CFIR framework	Prefers risk info at decision points	Not assessed	Barriers: ML opacity; facilitators: education, feedback
Ramgopal et al. [41]	Perception study	Qualitative interviews on pediatric AI-CDSSs	Clinician acceptance insights	Not assessed	Focus on sociotechnical adoption factors

Abbreviations: ML = machine learning; AUROC = Area Under ROC Curve; MCC = Matthews Correlation Coefficient; CBC = Complete Blood Count; QALY = Quality-Adjusted Life Year; CDS = clinical decision support; UI = User Interface; EHR = Electronic Health Record; SHAP = Shapley Additive Explanations; STEMI/NSTEMI = ST-Elevation/Non-ST-Elevation Myocardial Infarction; MACE = Major Adverse Cardiac Events; CFIR = Consolidated Framework for Implementation Research; RCT = Randomized Controlled Trial. ↑ = increase; ↓ = decrease.

## Data Availability

No new data were created or analyzed in this study. This systematic review is based entirely on previously published studies identified through searches of PubMed, Scopus, and Embase. All included studies are cited in the reference list.

## Supplementary Materials

The following supporting information can be downloaded at: https://www.mdpi.com/article/10.3390/healthcare14182922/s1, Table S1. Detailed Technical Characteristics of Included AI-CDSS Studies. Table S2. EHR Integration and Digital-Infrastructure Characteristics of Included AI-CDSS Records. Table S3. Additional Equity and Bias Characteristics of Included AI-CDSS Studies. Table S4. Mapping of Included Studies to Review Domains and Domain-Specific Analytic Contributions. Table S5. Reported Performance Metrics of Included AI-CDSS Studies, as Reported in the Primary Studies. Table S6. Methodological Families of Included Studies. Table S7. Multilevel Classification of Equity Engagement Across Included Studies. Table S8. Comparative Regulatory and Liability Frameworks for AI-CDSS as Software as a Medical Device. File S1: PRISMA 2020 checklist [57]. File S2: Database Search Strategies.

## Abbreviations

The following abbreviations are used in this manuscript:

ACEP	American College of Emergency Physicians
AI-CDSS	Artificial Intelligence-Driven Clinical Decision Support System
AMI	Acute Myocardial Infarction
AUC	Area Under the Curve
AUROC	Area Under the Receiver Operating Characteristic Curve
BERT	Bidirectional Encoder Representations from Transformers
CART	Classification and Regression Trees
CBC	Complete Blood Count
CDS	Clinical Decision Support
CDSS	Clinical Decision Support System
CFIR	Consolidated Framework for Implementation Research
DL	Deep Learning
EBM	Evidence-Based Medicine
ECG	Electrocardiogram
ED	Emergency Department
EHR	Electronic Health Record
EMR	Electronic Medical Record
ESI	Emergency Severity Index
FDA	Food and Drug Administration
GBM	Gradient Boosting Machine
GDPR	General Data Protection Regulation
HCV	Hepatitis C Virus
HIPAA	Health Insurance Portability and Accountability Act
ICU	Intensive Care Unit
IRB	Institutional Review Board
LASSO	Least Absolute Shrinkage and Selection Operator
LGBM	Light Gradient Boosting Machine
LIME	Local Interpretable Model-Agnostic Explanations
MACE	Major Adverse Cardiac Events
MCC	Matthews Correlation Coefficient
ML	Machine Learning
NLP	Natural Language Processing
NPV	Negative Predictive Value
NSTEMI	Non-ST-Elevation Myocardial Infarction
PAMA	Protecting Access to Medicare Act
PRISMA	Preferred Reporting Items for Systematic Reviews and Meta-Analyses
QALY	Quality-Adjusted Life Year
RCT	Randomized Controlled Trial
RMSE	Root Mean Squared Error
RSV	Respiratory Syncytial Virus
SHAP	Shapley Additive Explanations
STEMI	ST-Elevation Myocardial Infarction
SVM	Support Vector Machine
UTAUT	Unified Theory of Acceptance and Use of Technology
UTI	Urinary Tract Infection
XGB	Extreme Gradient Boosting (XGBoost)
