# Supplementary material for: Real-World Implementation and Evaluation of AI-Driven Clinical Decision Support in Emergency Medicine: A Systematic Review
Source: Healthcare (Basel). 2026 Sep 9;14(18):2922. doi: 10.3390/healthcare14182922 (PMC13606772; doi:10.3390/healthcare14182922)
Supplement: Supplementary file 1 [file healthcare-14-02922-s001.zip › AI_CDSS Supplementary Tables S1-S8.pdf]

Supplementary Table S1. Detailed Technical Characteristics of Included AI-CDSS Studies

NR = not reported in the included article; NA = not applicable to the study design.

Study	Input Variables	Model Architecture / Configuration	Training/derivation dataset	Calibration	Uncertainty estimation	Computational / deployment requirements
Akhlaghi et al., 2024	Unstructured emergency-department triage note.	Exact predictive architecture NR in this implementation article; previously developed NLP-based admission-prediction model.	Previously trained using approximately 10 years of St Vincent's Hospital ED triage-note data; the deployed model was not retrained during this evaluation.	NR.	Diagnostic-performance confidence intervals were reported.	Microsoft Azure Machine Learning, MLflow, Databricks, PySpark, Azure blob storage, and Power BI; Power BI refreshed every 15 minutes.
Huang et al., 2024	Demographics; triage vital signs; medical history; chief complaint; past history; present illness; structured numeric physiological variables transformed into narrative text using the study's TCND approach.	Pretrained transformer text-classification models (including BioClinicalBERT and other biomedical/clinical BERT variants); 10-fold cross-validation. PLM configuration included batch size 16, 4 epochs, and learning rate 2×10^-5.	Single-hospital ED dataset from Shuang-Ho Hospital, May 2017–December 2019; approximately 250,000 records were available before task-specific sampling.	NR.	performance confidence intervals were used for evaluation.	Intel Core i7-9700F 3.0 GHz (8 cores), 64 GB DDR4 RAM, 512 GB NVMe storage, NVIDIA RTX 3090 (24 GB VRAM); JupyterLab, Python 3.10.11, PyTorch 2.0.1.
Lin et al., 2025	CBC+DIFF-derived variables, including white-blood-cell and differential measures, neutrophil/lymphocyte percentages and ratios, and advanced neutrophil characteristics generated by the hematology analyzer.	Tabular supervised-ML pipeline comparing multiple classifiers; LightGBM was selected for the web-based AI-CDSS.	Tri-Service General Hospital; suspected pneumonia-induced sepsis and control records collected in 2023, with earlier months used for development and December 2023 used as temporal validation.	Brier score and Brier Skill Score were reported; Brier score of approximately 0.13 was reported for several top-performing models. No calibration slope/intercept reported.	NR	Web-based AI-CDSS hosted on the hospital's internal server.
Lupei et al., 2022	Demographics, comorbidities, home medications, and routinely available vital-sign variables.	LASSO-guided feature selection followed by multivariable logistic regression embedded in an EHR clinical decision-support workflow.	Derivation cohort of 1,469 SARS-CoV-2-positive adults; additional temporal and prospective cohorts were used for validation.	Calibration was evaluated in the real-time validation cohort; a calibration plot is provided in Supporting Figure S4, and the article discusses calibration limitations.	AUROC confidence intervals were reported.	Real-time EHR-integrated CDS across 12 hospitals. Hardware/software specifications NR.
Ali et al., 2016	Streaming ED chief complaints; syndromic counts; temporal/seasonal information; meteorological variables such as temperature, rainfall, and humidity for forecasting; geospatial information for outbreak mapping.	Service-oriented visual-analytics architecture integrating syndromic classification, temporal/spatiotemporal outbreak detection, support-vector-regression forecasting, and GIS visualization.	Infectious-disease surveillance data from 2011–2015. Explicit ML train/test partitioning is NR in the article.	NR.	NR.	Service-oriented/web-based surveillance architecture with GIS/large-display visual analytics.
Flores et al., 2024	Age, sex, body temperature, urine dipstick results, and flow-cytometry urinalysis variables.	Sex-stratified random-forest and neural-network models; concordant predictions from both models generated the clinical LIS output. Exact neural-network layer configuration NR.	8,692 ED cases from April 2018–December 2021 for retrospective model creation; 70/30 stratified development/validation split. A later cross-sectional cohort and pre/post clinical cohorts were used for implementation evaluation.	NR.	NR	Python 3.9 and scikit-learn preprocessing; SAS Enterprise Miner 14.3; Python communication with the CDSS integration engine and LIS..
Higgins et al., 2024	Dataset variables included age, prior/active service use, presenting problem, facility, First Nations status, referral source, marital status, arrival mode, gender, triage category, temporal variables, and disposition/referral-related fields as reported by the authors.	Multiple supervised classifiers; Explainable Boosting Machine implemented as an interpretable generalized additive boosting model; 80/20 train-test split.	26,681 ED mental-health presentations from January 2016–December 2021; final analytic sample 26,166 after listwise deletion; 80/20 train-test split.	NR.	NR	SQL, Python, Excel, Seaborn, scikit-learn, imbalanced-learn, and InterpretML were used.
Fujimori et al., 2022	Chief complaints, medical history, vital signs, and other numeric/text EHR information used by the aortic-dissection prediction model.	XGBoost model with 5-fold cross-validation generating real-time aortic-dissection risk probabilities and alert thresholds.	27,550 ED patients at a tertiary Japanese hospital were used for model development/validation.	NR.	No formal patient-level uncertainty method reported; AUROC was reported with a 95% CI.	Online AI-CDSS deployed for the experiment using Amazon Web Services; specific hardware/runtime requirements NR.
Hinson et al., 2022	39 prediction variables including age/sex, chief complaint, active ICD-10 problems, vital signs and trends, routine labs and inflammatory markers, SARS-CoV-2 status, and respiratory/oxygen-support requirements.	Two random-forest models (50 estimators; minimum leaf size 10) for 24-h critical-care need and 72-h inpatient-care need; probabilities converted to a 1–10 deterioration-risk level.	Retrospective derivation cohort: 21,452 adult ED encounters/18,810 unique patients; 67% training and 33% testing.	Brier scores: 0.080 for the critical-care model and 0.129 for the inpatient/acute-care model; calibration curves by risk-level group are in Supplementary Figure 1.	No formal patient-level predictive uncertainty interval reported. AUROC 95% CIs were calculated using DeLong's method.	Real-time EHR integration; analyses in Python 3.6 using scikit-learn and SHAP. Model-development/evaluation code was publicly released.
Liu et al., 2021	Chest-pain symptoms, 12-lead ECG signals, and high-sensitivity troponin I; follow-up ECG/troponin values could update alerts.	Previously developed deep convolutional ECG model combined with a rule-based multimodal alarm strategy using symptoms and high-sensitivity troponin; full base-model architecture is reported in the cited prior development article.	Included article reports an AI-strategy development cohort of 25,002 ED visits (August 2019–April 2020) and prospective validation cohort of 14,296 visits (May–August 2020). The base ECG DLM was developed previously.	NR.	NR	Real-time background server; ECG AI result available in <10 seconds and smartphone alerts integrated with the care pathway.
Kawamoto et al., 2024	Parent-reported questionnaire variables including respiratory symptoms and duration, age, body temperature/current or maximum temperature, month of visit, and baseline characteristics; full feature list is in Multimedia Appendix 1.	XGBoost selected after hyperparameter grid search and 10-fold cross-validation.	Yokohama City Municipal Hospital, January 2009–December 2015; final sample 4,174 children ≤24 months, including 619 RSV-positive cases.	Graphical calibration assessment and Hosmer-Lemeshow goodness-of-fit assessment using 10 groups.	1,000 bootstrap resamples were used for stability/optimism assessment of discrimination; no separate patient-level	R version 4.2.0.

Study	Input Variables	Model Architecture / Configuration	Training/derivation dataset	Calibration	Uncertainty estimation	Computational / deployment requirements
					uncertainty interval was reported.	
Mattay et al., 2023	Free-text imaging indication entered by the ordering provider plus additional patient/provider information referenced by the system; exact additional variables are not enumerated.	Commercial AI predictive-text matching module maps free text to structured indications or a ranked shortlist; proprietary predictive architecture NR.	NR	NR	Matching uses a correlation/similarity decision rule, but uncertainty estimates are not provided.	Epic EMR, Radiant RIS, and CareSelect Imaging CDS; AIPT/Predictive Text BPA integrated into ordering workflow.
Greenbaum et al., 2019	Free-text triage note plus routinely available ED variables used by the interface/model, including demographics and triage/vital-sign context as described in the study.	HaPPy domain-specific ontology paired with a multiclass support-vector machine using bag-of-words text features to rank the top five presenting-problem labels.	Approximately 97,000 triage notes were used to train the predictive interface; subsequent structured data were used for iterative refinement.	NR.	Model outputs included ranked predictions/probabilities, but no formal uncertainty-estimation framework was reported.	Custom ED user interface integrated with the local documentation workflow/EHR; hardware and detailed runtime requirements NR.
Barton et al., 2024	Extant EHR data for patients ≥65 years with an in-system primary-care provider; detailed predictor list is NR in this article.	Existing machine-learning fall-risk algorithm linked to an EHR discharge alert; underlying predictive architecture NR in this implementation article.	NR	NR.	NR.	Live EHR-integrated CDS. Specific software/hardware/runtime requirements NR.
Piñero de Plaza et al., 2025	Underlying RAPIDx clinical/biochemical inputs are not fully specified in this evaluation article; the broader RAPIDx program uses cardiac clinical and biomarker information.	Underlying RAPIDx predictive-model architecture NR in this human-centered implementation evaluation; the study evaluates the deployed system using PROLIFERATE_AI and Bayesian EKE methods.	NR/NA in this article because the underlying predictive model was developed in prior RAPIDx studies referenced by the authors.	NR	Bayesian EKE was used for human-centered evaluation, not as model prediction uncertainty.	Multisite workflow deployment/evaluation; specific computing hardware/software for the predictive engine NR. Authors discuss the need for EMR embedding and automated data entry.
van de Maat et al., 2019	Model-specific predictors such as age, respiratory rate, oxygen saturation, nasal flaring/dyspnea, fever, wheeze/rales, chest-pain/auscultatory findings, and biomarkers such as CRP/PCT depending on the original model; predictors are listed by model in Table 1.	No new model was trained; the study externally validated previously published multivariable logistic-regression equations and CART decision rules.	NA	The Discussion notes prevalence differences and the potential need for calibration/recalibration.	No formal patient-level uncertainty method reported; confidence intervals were reported for performance estimates.	NR/NA; no model-training compute was required/reported for this validation study.
Tylman et al., 2016	Vital-sign-monitor/ECG sensor data; optional external evidence such as age, sex, symptoms and medical history; authors discuss adding blood-analysis variables and other external data.	Expert-designed causal Bayesian network with conditional probability tables derived from clinical evidence; supports missing evidence and outputs condition probabilities.	No conventional ML training set. Network structure was based on medical knowledge/textbooks and physician verification; probability tables were populated from literature/epidemiologic evidence. Preliminary validation used 29 real patient cases.	Conventional calibration assessment NR. Probabilities/priors were manually specified; future automatic adjustment using patient databases and physician diagnoses was proposed.	BN itself represents probabilistic belief/conditional probability, but no separate epistemic/aleatoric uncertainty interval or calibration-based uncertainty method was reported.	Designed for real-time operation connected to a vital-sign monitor/standalone hardware system. Exact CPU/RAM specifications are NR in this article; prior system papers contain additional hardware details.
Castro Urda et al., 2025	Age 40–70 years, ED/hospital admission, blood-test availability, absence of prior HCV serology, anti-HCV result, and reflex HCV-RNA result.	Rule/workflow-based EHR decision-support engine implementing programmed HCV-screening actions; no trainable predictive-model architecture described.	NA	NA for a non-probabilistic rule/workflow engine.	NA.	Integration with CGM Selene/electronic medical record and laboratory workflow; hardware requirements NR.
Rabinovich et al., 2022	Chest X-ray images.	Combination of four deep-learning models, one for each chest-X-ray finding; detailed network architectures are NR in this article and are described in the cited development study.	NR	NR.	NR.	Integrated with the Electronic Health Record and Radiology Information System; 7,689 chest X-rays were processed during the study period. Hardware/runtime specifications NR.
Jiang et al., 2018	Temporal/calendar variables (hour/day/week/month, public holiday and adjacent days) plus meteorological variables such as maximum/mean/minimum temperature, dew point, relative humidity, cloud cover, rainfall, wind speed, and short-term weather changes.	Feed-forward deep neural network with multiple hidden layers and tanh activation; improved genetic-algorithm feature selection, Hogwild! parallel SGD, ADADELTA, L2 regularization, and dropout.	245,377 A&ED records from Hong Kong, July 2009–March 2011. Earlier observations were used for model/feature-selection training and the remaining period for out-of-sample testing.	NR	No prediction intervals or formal uncertainty-estimation method reported; repeated testing/robustness analyses were used.	Parallelized Hogwild! SGD and ADADELTA were used to accelerate training; exact hardware specifications NR.
Sax et al., 2022	Exact predictive variables NR in this article.	Previously developed ML-based acute-heart-failure risk model intended for EHR CDS; exact predictive architecture NR in this pre-implementation article.	NR	NR.	NR.	Because the tool was not yet deployed, the article discusses planned feasibility requirements: EHR build, score-calculation timing, missing-data handling, and clinician-facing display. Specific hardware/software NR.
Fishe, 2024	NA.	NA; editorial/commentary with no study-specific AI-CDSS model.	NA.	NA.	NA.	No study-specific compute requirement. Generic implementation requirements discussed include AI software/device purchase, EHR/system integration, periodic updates and maintenance, vendor support, and workforce training.

Study	Input Variables	Model Architecture / Configuration	Training/derivation dataset	Calibration	Uncertainty estimation	Computational / deployment requirements
Ramgopal et al., 2024	NA.	NA; qualitative clinician-perception study with no study-specific AI prediction model.	NA.	NA.	NA.	NA for a study-specific AI model.

Supplementary Table S2. EHR Integration and Digital-Infrastructure Characteristics of Included AI-CDSS Records

NR = not reported in the included article; NA = not applicable to the study design

Study	EHR/EMR/RIS/LIS integration	Interoperability standards (FHIR/HL7)	Data harmonization / standardization	Real-time data acquisition	Cybersecurity	Data governance	Vendor / platform implementation issues
Akhlaghi et al., 2024	Yes - admission-prediction AI was integrated into St Vincent's electronic emergency patient-management/EMR system.	NR	NR - deployment engineering was described, but no interoperability-oriented data harmonization/common data model was reported.	Yes - live post-implementation evaluation in routine ED practice.	NR - no technical cybersecurity controls were reported.	Ethics approval reported; formal operational data-governance processes NR.	Azure ML, MLflow, Databricks, PySpark, Azure blob storage, and Power BI were used; explicit vendor-related implementation barrier NR.
Huang et al., 2024	No - retrospective proof-of-concept; the authors state that the model had not yet been implemented in daily ED workflows.	NR	NR - structured/categorical data were transformed into narrative text (TCND), but this was not reported as interoperability/data harmonization across systems.	No - retrospective model-development/internal-validation study.	NR	Research ethics/Helsinki oversight reported; operational data governance NR.	NR
Lin et al., 2025	Web-based AI-CDSS hosted on the hospital's internal server; direct EHR embedding NR.	NR	NR - structured CBC+DIFF variables were used, but no common data model or cross-system mapping was reported.	NR - web deployment was reported, but live source-data acquisition/latency was not explicitly described.	NR	NR	Internal-server deployment reported; explicit vendor-related implementation challenge NR.
Lupei et al., 2022	Yes - model implemented in the EHR and displayed as CDS on the patient sidebar across a 12-hospital health system.	NR - no FHIR or HL7 implementation reported.	Yes/partial - model inputs were explicitly mapped to ICD-10 codes, pharmaceutical subclasses, RxNorm codes, and EHR flowsheets for vital signs.	Yes - a prospective real-time dataset was evaluated after EHR implementation.	NR	NR - formal operational data-governance processes were not described in the included article.	NR
Ali et al., 2016	Service-oriented surveillance architecture; direct EHR embedding NR.	NR	NR - heterogeneous data streams were integrated for analysis, but no interoperability-oriented harmonization standard/mapping was reported.	Yes - the system used real-time streaming ED data and automated data acquisition.	NR	NR	NR
Flores et al., 2024	Yes for laboratory workflow - predictions/comments were integrated into the LIS through a CDSS integration engine; direct EHR/EMR integration NR.	NR	NR - standardized laboratory variables were used, but no cross-system terminology/common-data-model harmonization was reported.	Yes - model calculations and LIS comments were generated automatically after urinalysis results became available.	NR	NR	Commercial LIS/CDSS components were used; explicit vendor-related implementation barrier NR.
Higgins et al., 2024	No live clinical integration - retrospective model-development/internal-validation study.	NR	NR	No - retrospective dataset.	NR	Ethics/site approval reported; operational data governance NR.	NR
Fujimori et al., 2022	Online pre-deployment/simulation AI-CDSS using EHR-derived numeric/text information; direct EHR embedding NR.	NR	NR - numeric/text data were organized with NLP, but no interoperability-oriented harmonization standard or mapping was reported.	Simulation only - the interface accepted real-time screen input and generated real-time risk output; live EHR feed/acquisition NR.	NR	Interview data were deidentified; operational governance of a deployed AI system NR.	Amazon Web Services was used to conduct the online experiment; explicit vendor-related barrier NR.
Hinson et al., 2022	Yes - model-generated deterioration risk levels were displayed in real time within the EHR disposition workflow.	NR	NR	Yes - real-time EHR-integrated CDS was prospectively deployed.	NR	NR	NR
Liu et al., 2021	Real-time ECG/background-server workflow with smartphone alerts; direct EHR embedding NR.	NR	NR - symptoms, ECG, and high-sensitivity troponin were combined, but no formal interoperability/harmonization method was reported.	Yes - ECG AI output was available in <10 seconds and subsequent ECG/troponin results could update alerts.	NR	IRB/Helsinki oversight reported; operational data governance NR.	NR
Kawamoto et al., 2024	No deployed AI-CDSS/EHR integration - retrospective model development using prospectively curated electronic-template data.	NR	Yes/partial - an automated electronic interview template was specifically introduced to standardize symptom entry; this is data-entry standardization, not cross-system interoperability.	No - the AI model was evaluated retrospectively; the template was completed before visits.	NR	Study-level privacy/ethics procedures reported; operational AI data governance NR.	NR
Mattay et al., 2023	Yes - commercial predictive-text functionality operated within Epic EMR/Radiant RIS/CareSelect Imaging CDS workflow.	NR	Yes - free-text imaging indications were mapped to standardized structured indications or a ranked structured shortlist.	Live point-of-ordering CDS interaction; source-data streaming/acquisition architecture NR.	NR	Regulatory/workflow controls were discussed; formal operational data governance NR.	Commercial/proprietary Epic/Radiant/CareSelect stack; explicit vendor lock-in/support barrier NR.
Greenbaum et al., 2019	Yes - the ML-assisted presenting-problem interface was fully integrated into the local clinical/EHR workflow; no additional data entry was required for model inputs.	NR for FHIR/HL7. SNOMED CT terminology was used within the HaPPy ontology, but HL7 appeared only in cited background literature rather than as an implemented standard.	Yes - HaPPy/SNOMED-based controlled vocabulary, term mapping, and iterative ontology refinement standardized presenting-problem documentation.	Yes/partial - live point-of-triage inputs were already collected in the clinical system; no separate streaming architecture was reported.	NR	NR	Custom/local interface; explicit vendor-related implementation barrier NR.
Barton et al., 2024	Yes - live EHR-integrated fall-risk CDS alert at ED discharge; the tool became a required hard stop after implementation changes.	NR	NR	Yes/partial - eligible patients were automatically assessed from extant EHR data and the alert fired during the live discharge workflow; source-data latency NR.	NR	Study ethics/qualitative procedures reported; operational data governance of the CDS NR.	NR
Piñero de Plaza et al., 2025	Multisite RAPIDx implementation/evaluation, but full EMR embedding was not established; manual data entry remained and authors identified EMR embedding/automation as future needs.	NR	NR	Real-time clinical use was discussed; automated source-data acquisition architecture NR.	NR	Human-centered implementation governance was evaluated, but formal operational data-governance controls NR.	Infrastructure and training burden were reported; explicit vendor-specific lock-in/support problem NR.

Study	EHR/EMR/RIS/LIS integration	Interoperability standards (FHIR/HL7)	Data harmonization / standardization	Real-time data acquisition	Cybersecurity	Data governance	Vendor / platform implementation issues
van de Maat et al., 2019	No AI-CDSS/EHR deployment - external validation of existing clinical prediction models; authors note these models had not been implemented in clinical practice.	NR	NR	No - prospectively collected validation data were analyzed, but there was no real-time deployed CDS acquisition workflow.	NR	Yes/limited - participant data were to be shared after de-identification under controlled, proposal-based access; operational AI governance was not applicable.	NR
Tylman et al., 2016	No EHR integration - stand-alone embedded device connected to a standard vital-sign monitor.	NR - FHIR/HL7 were not reported; the authors instead identified compatibility with multiple monitor models/data formats as an implementation requirement.	NR - signal preprocessing/data-format handling was described, but not interoperability-oriented harmonization.	Yes - automatic real-time acquisition/processing from the vital-sign monitor.	NR - networking/connectivity descriptions did not specify cybersecurity controls.	NR	Yes/partial - future deployment required compatibility with a wide range of vital-sign monitors; vendor lock-in/support was not specifically reported.
Castro Urda et al., 2025	Yes - CGM Selene electronic medical record, CGM DISCERN decision support, and laboratory/reflex-testing workflow were integrated.	NR	NR - the workflow linked screening, laboratory, and follow-up processes, but no explicit interoperability mapping/common data model was reported.	Automated event-driven workflow; explicit source-data latency/real-time acquisition architecture NR.	NR	Institutional/ethical oversight and multidisciplinary implementation were described; formal operational data-governance controls NR.	Commercial CGM platform involved; explicit vendor-related implementation barrier NR.
Rabinovich et al., 2022	Yes - TRx was integrated in the hospital's EHR and Radiology Information System (RIS).	NR	NR	Live real-world implementation; explicit real-time image acquisition/processing latency NR.	NR	NR	NR
Jiang et al., 2018	No live EHR/CDS deployment - retrospective A&ED patient-flow forecasting study.	NR	NR - temporal and meteorological inputs were combined analytically, but no interoperability/data-harmonization method was reported.	No - retrospective dataset/model evaluation.	NR	NR	NR
Sax et al., 2022	Planned EHR build/integration only; the tool had not yet been deployed in the study.	NR	NR - missing-data handling was a planned feasibility issue, but no harmonization method was reported.	Planned for real-time clinical workflows; actual real-time acquisition NR/not yet implemented.	NR	NR	NR
Fishe, 2024	Commentary only - EHR/system integration discussed as a generic implementation requirement; no study-specific deployment.	NR	NR	NA - no study-specific AI-CDSS deployment.	NR	Generic policy/equity issues were discussed; no study-specific operational data-governance implementation.	Yes, as commentary - costs of integration/maintenance, variability in vendor support, upgrade timing, tool failure, and company longevity were identified as implementation concerns.
Ramgopal et al., 2024	NA for a study-specific AI-CDS - qualitative clinician-perception study; the local pediatric CDS tools described did not use AI for underlying predictions.	NR/NA	NA	NA	NR	NR	NR

Abbreviations: AI-CDSS, artificial intelligence-driven clinical decision support system; CDS, clinical decision support; ED, emergency department; EHR, electronic health record; EMR, electronic medical record; FHIR, Fast Healthcare Interoperability Resources; HL7, Health Level Seven; IRB, institutional review board; LIS, laboratory information system; NR, not reported; NA, not applicable; RIS, radiology information system.

Supplementary Table S3. Additional Equity and Bias Characteristics of Included AI-CDSS Studies

NR = not reported in the included article; NA = not applicable to the study design.

Study	Demographic fairness / subgroup performance	Socioeconomic factors / disparities	Racial / ethnic bias	Dataset representativeness
Akhlaghi et al., 2024	Acknowledged but not evaluated; authors stated that inequity in Indigenous patients and gender bias could not be assessed.	NR for patient-level socioeconomic variables/fairness.	Indigenous subgroup inequity was explicitly identified as unassessed; no racial/ethnic subgroup performance analysis was reported.	The original model excluded short-stay admission data; authors called for larger, more varied datasets and retraining. No formal representativeness audit was reported.
Huang et al., 2024	NR; no demographic subgroup performance/fairness analysis identified.	NR.	NR.	Approximately 250,000 records were available from one ED before task-specific sampling; no explicit demographic representativeness/fairness audit was reported.
Lin et al., 2025	NR; no demographic subgroup fairness/performance analysis identified.	Resource constraints were discussed at the health-system/country level, not as patient-level socioeconomic fairness.	NR.	Local 2023 hospital data were used for development/temporal validation; no formal representativeness audit was reported.
Lupei et al., 2022	Yes. Explicit sensitivity analyses compared performance across gender and racial/ethnic groups using AUROC, 95% CIs, and p-values.	Socioeconomic status was not directly evaluated as a subgroup; authors noted the need for validation in populations with different socioeconomic backgrounds.	Yes. Race/ethnicity were included and performance was compared across White, Black, Asian, Hispanic, Declined, and Other groups; authors warned that race-unaware models may reflect the majority population.	Authors explicitly discussed majority/minority representation and the risk of underestimating minority risk; validation was limited to one regional health system.
Ali et al., 2016	NR for demographic subgroup performance/fairness.	NR.	NR.	Evaluation used infectious-disease surveillance data from 2011–2015 in Pakistan; no demographic representativeness audit was identified.
Flores et al., 2024	Yes/partial. Predictive models were divided by gender and model performance was evaluated by gender.	NR.	NR.	Pregnant women and patients aged <=12 years were excluded; the development sample came from one local ED population.
Higgins et al., 2024	Conceptual concern only; potential bias/discrimination in vulnerable mental-health populations was discussed, but no demographic subgroup performance analysis was reported.	NR for explicit socioeconomic fairness; marital status/service-use variables were included, but no socioeconomic subgroup fairness test was reported.	First Nations status was included as a variable/context; no racial/ethnic subgroup performance or formal fairness evaluation was reported.	The dataset included 26,166 mental-health presentations from one Local Health District; vulnerable-population bias was discussed, but representativeness was not formally audited.
Fujimori et al., 2022	NR for patient demographic subgroup fairness/performance.	NR.	NR.	Model development/validation used one tertiary Japanese hospital, and the implementation/usability experiment included 14 physicians from two hospitals; no demographic representativeness audit was identified.
Hinson et al., 2022	Demographic representation was reported, but no demographic subgroup performance/fairness test was identified.	NR.	Race/ethnicity composition was reported across cohorts, but no racial/ethnic performance comparison was reported.	Cohorts included representation across age, gender, race, and ethnicity, including White non-Latino, Black non-Latino, and Latino patients; no subgroup fairness analysis was performed.
Liu et al., 2021	NR; no demographic subgroup fairness/performance analysis identified.	NR.	NR.	Large development and prospective validation cohorts were reported at the same center, but no explicit demographic representativeness/fairness audit was identified.
Kawamoto et al., 2024	NR; no demographic subgroup fairness/performance analysis identified.	NR.	NR.	The final sample included 4,174 children <=24 months from one municipal hospital; no demographic representativeness/fairness audit was identified.
Mattay et al., 2023	NR; no demographic subgroup fairness/performance analysis identified.	NR.	NR.	Training/derivation data for the proprietary predictive-text model were not reported, limiting assessment of dataset representativeness.
Greenbaum et al., 2019	NR for demographic subgroup fairness; a reported 'subgroup analysis' concerned system downtime rather than demographic groups.	NR.	NR.	Patients bypassing triage, often the sickest patients, were excluded; authors performed a sensitivity analysis reincluding all 811 excluded patients.
Barton et al., 2024	NR in this implementation article.	NR.	NR.	Eligibility was restricted to patients >=65 years with an in-system primary-care provider; underlying model-development data were reported in prior studies, not this article.
Piñero de Plaza et al., 2025	Patient-level algorithmic fairness was not directly evaluated; user experience was assessed across diverse clinical roles and experience levels.	Patient-level socioeconomic fairness NR.	NR	Clinician/stakeholder recruitment used stratified random sampling to reduce selection bias and improve representation across roles/demographics; the underlying predictive-model training population was not fully specified.
van de Maat et al., 2019	NR for demographic subgroup fairness.	NR.	NR.	Authors described heterogeneous validation populations, differences from derivation populations, and prevalence differences that could affect predictions/generalizability.
Tylman et al., 2016	Sex and age were incorporated into Bayesian-network priors, but no demographic subgroup performance/fairness evaluation was conducted.	NR.	NR.	Authors identified a mismatch between whole-population morbidity rates and the Accident & Emergency population; sex/age priors were constructed from population data.
Castro Urda et al., 2025	NA/NR for algorithmic demographic fairness because the intervention was a rule/workflow-based HCV screening pathway rather than a trainable predictive model.	Access-to-care issues were discussed because some patients do not regularly see healthcare providers, but no socioeconomic subgroup analysis was reported.	NR.	The target population was restricted by workflow eligibility, including age 40–70 years; no predictive-model training dataset was applicable.
Rabinovich et al., 2022	NR in this implementation/user-satisfaction article.	NR.	NR.	The study processed 7,689 chest X-rays, but the training populations for the four underlying deep-learning models were not reported in this included article.

Study	Demographic fairness / subgroup performance	Socioeconomic factors / disparities	Racial / ethnic bias	Dataset representativeness
Jiang et al., 2018	NR/NA for patient-level demographic fairness because the model forecasts aggregate A&E patient flow rather than individual clinical risk.	NR.	NR.	The model used 245,377 A&E records from Hong Kong; no demographic representativeness audit was reported.
Sax et al., 2022	Stakeholder concern only; no empirical demographic subgroup performance analysis was reported.	No direct patient-level SES analysis; one clinician requested comparison of KPNC members versus non-members, reflecting a potential access/system-membership concern.	Clinicians raised concerns that input biases could affect equity across patient populations; no racial/ethnic subgroup evaluation was reported.	Underlying model-development data were not reported in this article; clinicians explicitly raised concerns that outputs could reflect biases in how the dataset was created or coded.
Fishe, 2024	Conceptual discussion only; no empirical model/subgroup performance study.	Yes, at an institutional/resource level: unequal financial and academic capacity to adopt AI was discussed, especially for rural and under-resourced EDs.	Yes, conceptually: race/ethnicity differences in presentation/triage and concern that homogeneous training populations may not generalize to diverse populations were discussed.	Strong conceptual concern was raised about homogeneous training/validation populations and applicability to diverse or underrepresented ED populations.
Ramgopal et al., 2024	NA for patient algorithmic fairness; qualitative clinician-perception study with no study-specific AI prediction model tested.	NR.	NR.	Clinician-perception sample only; no model training/validation dataset was evaluated in this study.

Note: Formal fairness metrics (e.g., demographic parity, equalized odds/equal opportunity, disparate impact, or equivalent fairness-specific statistics) were not reported by any included empirical study and therefore are not included as a separate column. Geographic variability/generalizability is summarized in the main manuscript tables, and reported bias-mitigation strategies are summarized in Table 3.

Demographic fairness/subgroup performance was coded only when the article explicitly evaluated demographic subgroups or explicitly discussed the inability to do so. Dataset representativeness reflects reported sampling, eligibility, or population limitations and should not be interpreted as proof of bias.

Supplementary Table S4. Mapping of Included Studies to Review Domains and Domain-Specific Analytic Contributions

This table maps each of the 23 included publications to the thematic analyses used in the review. "Yes" indicates contribution to the specified domain-specific synthesis; "No" indicates that the publication remained part of the overall review but did not contribute to that domain because its design, focus, or reporting did not provide the information used for that synthesis.

Study	Technical characteristics / Figure 2	Clinical applications / Section 3.2	Implementation dimensions / Section 3.3	Organizational, policy, and ethical / Section 3.4	Real-world evaluation / Section 3.5	Reason for domain-specific non-contribution
Akhlaghi et al., 2024	Yes	Yes	Yes	Yes	Yes	-
Huang et al., 2024	Yes	Yes	Yes	Yes	Yes	-
Lin et al., 2025	Yes	Yes	Yes	Yes	Yes	-
Lupei et al., 2022	Yes	Yes	Yes	Yes	Yes	-
Ali et al., 2016	Yes	Yes	Yes	Yes	Yes	-
Flores et al., 2024	Yes	Yes	Yes	Yes	Yes	-
Higgins et al., 2024	Yes	Yes	Yes	Yes	Yes	-
Fujimori et al., 2022	Yes	Yes	Yes	Yes	Yes	-
Hinson et al., 2022	Yes	Yes	No	Yes	Yes	Section 3.3: The publication was synthesized primarily as a prospective real-world performance/effectiveness evaluation and was not included in the standardized implementation-dimensions subset summarized in Table 2.
Liu et al., 2021	Yes	Yes	Yes	Yes	Yes	-
Kawamoto et al., 2024	Yes	Yes	No	No	No	Section 3.3: Retrospective model-development/validation study without clinical workflow deployment or implementation evaluation. Section 3.4: No substantive organizational, governance, policy, or ethical implementation analysis. Section 3.5: Treated as a model-development/validation study rather than a real-world implementation/effectiveness evaluation.
Greenbaum et al., 2019	Yes	Yes	Yes	Yes	Yes	-
Tylman et al., 2016	Yes	Yes	Yes	Yes	Yes	-
van de Maat et al., 2019	Yes	Yes	No	Yes	Yes	Section 3.3: External model-validation study without clinical AI-CDSS deployment, workflow integration, usability, or implementation evaluation.
Mattay et al., 2023	Yes	Yes	Yes	Yes	Yes	-
Castro Urda et al., 2025	Yes	Yes	Yes	Yes	Yes	-
Rabinovich et al., 2022	Yes	Yes	No	No	Yes	Section 3.3: The publication focused on post-deployment user satisfaction rather than the broader implementation dimensions summarized in Table 2. Section 3.4: Primarily a usability/user-satisfaction evaluation rather than an organizational, policy, regulatory, or equity analysis.
Jiang et al., 2018	Yes	Yes	Yes	Yes	No	Section 3.5: Retrospective patient-flow forecasting using historical A&ED data; the study did not evaluate the effects of an implemented AI-CDSS on clinical workflow, users, or patient outcomes.
Sax et al., 2022	No	No	Yes	Yes	Yes	Technical/Figure 2: Pre-implementation study centered on implementation readiness around a previously developed ML model rather than study-specific technical characterization. Section 3.2: No study-specific clinical use of the AI tool was evaluated before deployment.
Barton et al., 2024	Yes	Yes	Yes	Yes	Yes	-
Pinero de Plaza et al., 2025	Yes	Yes	Yes	Yes	Yes	-
Fishe, 2024	No	No	Yes	Yes	No	Technical/Figure 2: Editorial/perspective without study-specific model development or technical evaluation. Section 3.2: No study-specific clinical AI-CDSS application was evaluated. Section 3.5: No empirical cohort or real-world outcome evaluation; the publication contributed conceptual implementation, policy, vendor, and equity considerations.
Ramgopal et al., 2024	No	No	No	No	Yes	Technical/Figure 2 and Section 3.2: Qualitative clinician-perception study without a study-specific AI prediction model or AI-CDSS clinical application. Section 3.3: Focused on general clinician perspectives rather than implementation of a specific AI-CDSS. Section 3.4: Used in the human-centered evaluation synthesis rather than the organizational/policy/ethical synthesis.

Note. Domain-specific non-contribution should not be interpreted as exclusion from the systematic review. The thematic domains were not mutually exclusive, and individual publications could contribute to more than one domain. The analytic subsets used in the manuscript are: technical characteristics/Figure 2, 20 of 23; clinical applications/Section 3.2, 20 of 23; implementation dimensions/Section 3.3, 18 of 23; and real-world evaluation/Section 3.5, 20 of 23.

Supplementary Table S5. Reported Performance Metrics of Included AI-CDSS Studies, as Reported in the Primary Studies

Metrics are presented in the units used by each primary study. "Not reported" indicates the metric was not reported in the source. Values are not pooled or compared across studies because target conditions, outcome definitions, case-mix and prevalence differ. Only 8 of 20 studies with an evaluable model reported an AUROC.

Study	Algorithm(s)	Primary metric type	Discrimination / primary result	Sensitivity / Specificity	External validation	Interpretability method	Model updating
Higgins et al. [22]	RF, XGB, CatBoost, kNN, EBM (InterpretML), SVM	AUROC (+MCC)	EBM 0.717; RF 0.682; CatBoost best by MCC 0.20	Sens 0.108 / Spec 0.981 (EBM)	Single site; no external validation	EBM interpretable; SHAP proposed	Maintenance not specified
Flores et al. [21]	RF, NN	AUROC	RF 0.80; NN 0.77; implementation stage AUC 0.81–0.88	Not reported	Single-site validation	No SHAP/LIME	Not reported
Lupei et al. [19]	Logistic regression (LASSO-logit)	AUROC	Temporal 0.87; real-time 0.85; range 0.82–0.87	Not reported	Multi-site (12 hospitals, same system)	Interpretable coefficients	Calibration issues; update implied
Tylman et al. [28]	Bayesian network, ECG segmentation	Sensitivity/Specificity	No AUROC reported	Sens 0.53–0.68 / Spec 0.90–1.00	29 real patient cases	BN probabilities interpretable	Auto-adjust via physician input
Barton et al. [33]	ML-based fall risk prediction algorithm	Qualitative	No model metric (implementation study)	Not applicable	Single academic medical center (Wisconsin)	None discussed in implementation study	Not reported
Greenbaum et al. [27]	HaPPy ontology, multiclass SVM	Accuracy	Best-5 accuracy 0.757	Not reported	Single tertiary center	Not detailed	Iterative ontology refinement
Kawamoto et al. [26]	XGBoost	AUROC	0.72–0.78	Sens/Spec ≈74%	Single cohort	SHAP for feature importance	Not reported
Jiang et al. [34]	Deep neural network	RMSE / MAPE	Values not stated in source	Not applicable	Single-site	GA feature selection	Not reported
Hinson et al. [24]	RF, CART	AUROC	0.91→0.85 (CCU); 0.89→0.80 (inpatient)	Not reported	Single health system	Decision trees interpretable	Not reported
Ali et al. [20]	kNN, geospatial (SaTScan)	NRMSE / accuracy	Syndromic classification >95% accuracy; dengue forecast NRMSE 0.29	Not reported	Not reported	Not reported	Not reported
Lin et al. [18]	LDA, LR, RF, GBM, AdaBoost, XGB, LGBM	AUROC	LGBM 0.90	Sens 83% / Spec 85%	Limited; multicenter needed	SHAP + heatmaps	Not reported
van de Maat et al. [29]	Logistic regression, CART	ORC	7 models; 3 met ORC ≥0.55	Sens/Spec reported, values not extracted	Dual-site (NL + UK)	CART transparent; LR interpretable	Not reported
Akhlaghi et al. [16]	NLP-based ML	Accuracy / Sens / Spec	Accuracy 83%→74% post-deployment	Sens 73.1% / Spec 74.3%	Single-site	Binary outputs; no rationale	Not reported
Piñero de Plaza et al. [12]	RAPIDx AI (black-box)	None (human-centred)	No model performance metric reported	Not applicable	Multi-site (12 EDs)	Numeric outputs only	Not reported
Mattay et al. [30]	Predictive text AI module	Process metrics	Unscored orders 81%→45%; +24 s interaction time	Not applicable	Single quaternary ED	Ranked shortlist; no SHAP	Not reported
Huang et al. [15]	NLP, ML/DL, BioClinicBERT variants	AUROC	BioClinicalBERT 0.90–0.92 across tasks	Not reported	Single hospital (Taiwan)	Not reported	Not reported
Fujimori et al. [23]	XGBoost (5-fold CV)	AUROC	XGBoost 0.901 (95% CI 0.84–0.96)	Not reported	Small sample; simulated	SHAP bar charts	Not reported
Liu et al. [25]	Deep learning	F1	AMI 0.85; STEMI 0.93; NSTEMI 0.70	Not reported	Single academic center	Not reported	Not reported
Rabinovich et al. [32]	AI for chest X-ray interpretation	Satisfaction	No model metric (survey study)	Not applicable	Not specified	Focus on clinician trust evaluation	Not reported
Castro Urda et al. [31]	AI-based Intelligen-C strategy for HCV screening	Process / economic	8× serology increase; €31/QALY; +2.5 QALYs	Not applicable	Single health system	Not reported	Not reported

Supplementary Table S6. Methodological Families of Included Studies

All 23 included studies are classified into exactly one family. Reported performance is reproduced as extracted and is not comparable across families; no included study compared families head-to-head on a shared cohort.

Methodology family	n studies	Studies
Conventional and other non-deep ML / probabilistic models	11	Lin et al. [18]; Lupei et al. [19]; Ali et al. [20]; Flores et al. [21]; Higgins et al. [22]; Fujimori et al. [23]; Hinson et al. [24]; Kawamoto et al. [26]; Greenbaum et al. [27]; Tylman et al. [28]; van de Maat et al. [29]
Deep neural networks (non-transformer)	2	Liu et al. [25]; Jiang et al. [34]
Transformer / pre-trained language models	1	Huang et al. [17]
NLP without specified deep architecture	1	Akhlaghi et al. [16]
Previously developed / proprietary model; architecture not reported in the included article	5	Piñero de Plaza et al. [12]; Mattay et al. [30]; Rabinovich et al. [32]; Barton et al. [33]; Sax et al. [35]
Reinforcement learning	0	—
Federated learning	0	—
Large language models (generative)	0	—
No study-specific trainable AI model evaluated	3	Castro Urda et al. [31]; Fishe [36]; Ramgopal et al. [41]

Supplementary Table S7. Multilevel Classification of Equity Engagement Across Included Studies

Levels follow the framework introduced in Section 3.4.3: algorithmic fairness, procedural equity and distributive equity. Basis column reproduces the extracted evidence supporting each classification.

Study	Equity level	Engagement	Basis in extracted data
Higgins et al. [22]	Algorithmic fairness	Acknowledged only	Flags bias risk when applying ML to mental-health populations where vulnerable subgroups are over-represented; no subgroup analysis performed.
Tylman et al. [28]	Algorithmic fairness	Implemented	Subgroup analysis across gender and race; Bayesian-network root probabilities set by admission proportion.
Huang et al. [17]	Algorithmic fairness	Implemented	Balanced statistical sampling and cross-validation to counter distributional skew in NLP training corpus.
Lupei et al. [19]	Algorithmic fairness	Implemented	Performance reported as fair across gender and race (Table 4); severe-COVID subgroup disparity 0% vs 33% documented.
Hinson et al. [24]	Algorithmic fairness	Implemented (indirect)	Calendar and meteorological covariates incorporated to reduce temporal confounding; no demographic subgroup analysis.
Ali et al. [20]	Algorithmic fairness	Implemented (indirect)	Demographic and clinical features incorporated into syndromic classification; representativeness not formally evaluated.
Greenbaum et al. [27]	Algorithmic fairness	Limitation acknowledged	Single-centre ontology and model noted to limit generalizability; no fairness evaluation.
Flores et al. [21]	Algorithmic fairness	Not addressed	Limited ethical discussion; no fairness metric or subgroup analysis reported.
Liu et al. [25]	Algorithmic fairness	Not addressed	No fairness assessment or mitigation reported despite prospective deployment.
Fujimori et al. [23]	Procedural equity	Implemented	SHAP explanations deployed to reduce clinician anchoring bias; alert thresholds tuned to limit alert fatigue.
Piñero de Plaza et al. [12]	Procedural equity	Implemented	Disparities in clinician confidence and output interpretation across experience levels; RAPIDx AI adapted to variation in clinical expertise across 12 EDs.
Sax et al. [35]	Procedural equity	Acknowledged only	Bias transparency emphasised in pre-implementation interviews; no formal governance or mitigation plan.
Akhlaghi et al. [16]	Procedural equity	Acknowledged only	Privacy, consent and justice raised; protections limited to general research-ethics oversight.
Barton et al. [33]	Procedural equity	Implemented	Academic detailing as implementation strategy; addressed clinician misconceptions in referral pathway (workforce readiness).
Fishe [36]	Distributive equity	Advocated	Under-resourced and rural EDs lack financial and legal capacity to implement AI responsibly; risk of digital marginalisation.
Lin et al. [18]	Distributive equity	Acknowledged only	Limited data availability in developing countries allows models trained on non-representative populations to reinforce inequities.
Castro Urda et al. [31]	Distributive equity	Implemented (indirect)	Child-centred design reduces procedural discomfort; €31/QALY cost-effectiveness supports scalability to resource-variable settings.
Jiang et al. [34]	Distributive equity	Implemented (indirect)	Patient-flow modelling across severity levels to support equitable resource allocation.

Supplementary Table S8. Comparative Regulatory and Liability Frameworks for AI-CDSS as Software as a Medical Device

Position as at August 2026. Instruments are cited by title and date in the reference list; the EU Product Liability Directive transposition deadline of 9 December 2026 had not passed at the time of writing.

Dimension	United States	European Union	China
Primary device pathway	FDA SaMD framework; 510(k) / De Novo / PMA. Clinical decision support meeting the 21st Century Cures Act §3060 criteria may fall outside device regulation where the clinician can independently review the basis of the recommendation.	Medical Device Regulation (EU) 2017/745 conformity assessment; most diagnostic/triage AI-CDSS classify as Class IIa or higher, requiring notified-body involvement.	NMPA registration under the Medical Device Supervision and Administration Regulations; AI software reviewed via CMDE technical review guidance for AI-based medical device software.
AI-specific overlay	Predetermined Change Control Plan (PCCP) final guidance (December 2024) permits pre-authorised model updates; draft guidance on AI-enabled device software functions (January 2025) addresses total product lifecycle documentation and transparency.	AI Act, Regulation (EU) 2024/1689 — AI used as a medical device safety component is high-risk, triggering risk management, data governance, logging, human oversight and post-market monitoring obligations layered on MDR.	CMDE guidance lineage from the 2019 deep-learning-assisted decision software review points to the 2022 AI medical device software guideline; emphasis on algorithm traceability, dataset quality and lifecycle change control.
Civil liability route	State tort law: medical malpractice against the clinician (standard of care), product liability against the manufacturer. The learned-intermediary doctrine and the absence of a federal AI liability statute concentrate exposure on the treating physician.	Revised Product Liability Directive (EU) 2024/2853 brings standalone software and AI within strict product liability, adds disclosure duties and rebuttable presumptions of defect/causation for technically complex products; Member State transposition due 9 December 2026.	Civil Code product-liability and medical-damage provisions; the medical institution is the primary defendant for treatment injury, with recourse against the manufacturer for defective product.
Status of a dedicated AI liability instrument	None enacted; no federal cause of action specific to AI.	The proposed AI Liability Directive was withdrawn by the Commission; the revised PLD is now the principal harmonised route.	No dedicated AI liability statute; addressed through existing Civil Code and sectoral regulation.
Practical allocation for an already-licensed tool	Regulatory clearance evidences reasonable design but does not discharge the physician's independent duty; deviation from the standard of care remains the operative test.	CE marking and AI Act conformity do not immunise the manufacturer under the revised PLD; the deployer retains obligations for human oversight and use in accordance with instructions.	Registration approval is a market-entry condition; clinical responsibility remains with the institution and treating clinician.
