# Supplementary material for: Real-World Implementation and Evaluation of AI-Driven Clinical Decision Support in Emergency Medicine: A Systematic Review
Source: Healthcare (Basel). 2026 Sep 9;14(18):2922. doi: 10.3390/healthcare14182922 (PMC13606772; doi:10.3390/healthcare14182922)
Supplement: Supplementary file 1 [file healthcare-14-02922-s001.zip › File S2. Search Strategies.pdf]

Database Search Strategies

PubMed

("Emergency Service, Hospital"[Mesh] OR Emergency-room* OR Emergency-department* OR Emergency-unit* OR Emergency-service OR Emergency ward)
AND
("Artificial Intelligence"[Mesh] OR "Artificial Intelligence" OR machine-learning OR machine-intelligence OR deep-learning OR LLM OR Large-Language-model OR data-mining OR prediction-model* OR prediction-analytic)
AND
(decision-support-system* OR "Decision Support Systems, Clinical"[Mesh])
AND
(implemented OR implementation* OR deployment [tiab])
Last 10 years
Results: 167 studies

Scopus

(Emergency-room* OR Emergency-department* OR Emergency-unit* OR Emergency-service OR Emergency ward)
AND
("Artificial Intelligence" OR machine-learning OR machine-intelligence OR deep-learning OR LLM OR Large-Language-model OR data-mining OR prediction-model* OR prediction-analytic)
AND
(decision-support-system*)
AND
(implemented OR implementation* OR deployment)
Limited to 2015–2025, article and review papers
Results: 149 studies

Embase

('hospital emergency service'/exp OR Emergency-room* OR Emergency-department* OR Emergency-unit* OR Emergency-service OR Emergency ward)
AND
('artificial intelligence'/exp OR "Artificial Intelligence" OR machine-learning OR machine-intelligence OR deep-learning OR LLM OR Large-Language-model OR data-mining OR prediction-model* OR prediction-analytic)
AND
(decision-support-system* OR 'clinical decision support system'/exp)

AND
(implemented OR implementation* OR deployment)
AND ('article'/it OR 'article in press'/it OR 'review'/it)
AND [2015–2025]/py
Results: 115 studies
